# Supplementary material for: Harnessing Tumor‐Specific Transcript Diversity Uncovers a Shared Neoantigen Reservoir for Pancreatic Ductal Adenocarcinoma
Source: MedComm (2020). 2026 Jul 30;7(8):e70890. doi: 10.1002/mco2.70890 (PMC13419689; doi:10.1002/mco2.70890)
Supplement: Supplementary file 1 — Supporting Information: mco270890‐supp‐0001‐SuppMat.pdf [file MCO2-7-e70890-s001.pdf]

Supplementary Materials for

Harnessing Tumor-Specific Transcript Diversity Uncovers a Shared Neoantigen Reservoir for Pancreatic Ductal Adenocarcinoma

Jingjing Zhao[1,2,#], *Qiaojuan Li*[1,2,#], *Peng Lin*[1,2,#], *Yu Yang*[1,2,], *Hongwu Yu*[1,2], *Yifan Wen*[1,2], *Wenqian Yu*[1,2], *Huiyi He*[1,2], *Sichen Tao*[1,2], *Feifei Zhang*[1,2], *Yan Li*[1,2], *Zhixiang Hu*[1,2], *Jing Xie*[1,2*], *Zhen Chen*[1,2,*], *Shenglin Huang*[1,2,3,4*]

Correspondence to: slhuang@fudan.edu.cn

Materials and Methods
Figures. S1 to S6
Tables S1 to S7

Materials and Methods

Splicing junctions classification

Splicing junctions classification was performed using a hierarchical two-step approach with bedtools[1] intersect under strand-specific matching (-s). In the primary annotation, each splicing junction was compared against GENCODE-annotated exonic ("E") and intronic ("I") regions. Splicing junctions where both donor and acceptor sites resided entirely within the same annotated exon or intron were assigned "E" or "I" labels, respectively; these labels were concatenated to form composite annotations, and all splicing junctions absent in GENCODE were flagged with "AS" (e.g., "I-I-AS" for intron alternative splicing junction).

Unannotated splicing junctions underwent secondary analysis: donor and acceptor positions were independently compared to genomic features. Positions overlapping exons received "E", introns "I", and non-overlapping sites "intergenic". Composite labels concatenated these assignments (e.g., "E-intergenic"). To simplify, we defined splicing junction types as follows: ‘E-E’ as normal, ‘E-E-AS’ as E-E-AS, ‘E-E-IAS’ as E-E-IAS, ‘E-I-AS’, ‘I-E-AS’, ‘I-I-AS’ and ‘I-I-IAS’ as intron alternative splicing

(intron_AS), and other splicing junctions were classified as intergenic splicing (E-intergenic, I- intergenic, intergenic-intergenic, intergenic-E, intergenic-I).

Chimeric-intergenic junctions were characterized by four splicing junction subtypes: E-intergenic, I-intergenic, intergenic-E, and intergenic-I junctions. In contrast, non-chimeric intergenic junctions specifically resulted from intergenic-intergenic junctions, where both splice sites resided within non-annotated intergenic regions. The genomic coordinates of transposable elements (TEs) were retrieved from the UCSC database (hg38). We implemented distinct genomic coordinate extraction strategies for different splicing event types: (1) full genomic coordinates for cryptic exons; (2) upstream exonic coordinates only for other intronic aberrant splicing junctions; (3) upstream and downstream exonic coordinates for chimeric intergenic junctions; and (4) both upstream and downstream exonic coordinates for non-chimeric intergenic junctions. TE overlap analysis was performed using bedtools intersect -wo command, which quantified TE coverage length for each exonic region. The TE with maximal coverage length was selected as the final annotation for junctions (refer to Figure. 3D).To identify overlaps between single-exon neoTSTs and TEs in the genome, the bedtools intersect (-wo) function was employed (refer to Figure. S3).

Transcription start sites annotation and regulatory analysis of F-neoTSTs

The bedtools intersect -s -wo command was used to compare transcription start sites (TSS) between F-neoTSTs and known transcripts. A novel TSS was defined if the TSS of F-neoTST was more than ±100 bp away from the TSS of any known transcript annotated by GENCODEv29. Histone modifications around the TSS regions (TSS ± 1000 bp, excluded chrM and chr_random) of F-neoTST were analyzed using the ChIP-Atlas Enrichment Analysis tool with pancreas cell type class (https://chip-atlas.org/enrichment_analysis). The result of the top 200 were visualized.

ChIP-seq peak data for 44 transcription factors (TFs) in pancreas cell type were obtained from ChIP-Atlas (https://chip-atlas.org/peak_browser). Using bedtools intersect with a 10% reciprocal overlap requirement (-f 0.1 -wo), we calculated the number of TSS regions (±1000 bp, excluding chrM and chr_random) regulated by each TF. TFs with a binding ratio (number of binding TSS/total TSS) were calculated and selected Top 10 for further visualization.

Identification of sc-neoTST in scRNA-seq

Single-cell RNA-seq of six PDAC panties tumor and NATs were downloaded from GEO under accession GSE212966. Cellranger (v6.0.0) and Seurat[2] (v4.0.5) were performed to obtain the cluster annotation (see *Single-cell RNA-seq analysis* section). The SCASL method[3] was used to identify splicing junctions in scRNA-seq data. We only analyzed cells with more than 2,000 genes to minimize issues related to sequencing depth. For each tumor samples, BAM file for each single cell were extracted, and the extract.py function in SCASL package was used to obtain splicing junctions for each single cell. For adjacent samples, splicing junctions were obtained for each sample directly, so no BAM file splitting was required.

To identify neoTST in tumor single cell (sc-neoTSTs), we filtered the splicing junctions (read count>1 via SCASL) identified in tumor single cells as follows: 1) Junctions identified in the matched adjacent tissue samples were excluded. 2) Junctions detected in GTEx datasets were excluded. 3) The remaining junctions were intersected with the junction derived neoTSTs identified in this study and those common to both were considered junction and its derived neoTST expressed at the single-cell level.

The feature of cells expressed sc-neoTSTs were evaluated by gene signature score through the AddModuleScore function in Seurat. Signature genes of fibroblast were downloaded from Gao et al[4]. Gene sets for EVs related pathway were downloaded from Gene Ontology and Reactome.

Identification of neoTSTs in EVs

Transcriptomes of PDAC extracellular vesicles (EVs) were in house[5-7]. Splicing junctions in EVs were comprehensively identified using ASJA with default parameters.

To systematically investigate the role of RNA-binding proteins (RBPs) in promoting neoTST enrichment in EVs, we integrated 111 ENCODE eCLIP-seq datasets (K562 cells; BED format). RBP binding sites were mapped to exonic regions of EV-associated neoTSTs (EV-neoTSTs) using bedtools intersect with strand-specific matching (-s -wo). Tissue-derived neoTSTs (Tissue-neoTSTs) served as the reference group. Background distributions were established through 100 iterations of random sampling from the tissue-neoTST pool. RBP binding frequencies between EV-neoTSTs and tissue-neoTSTs were statistically compared using Wilcoxon rank-sum tests. Key regulatory RBPs were prioritized by enrichment fold-change (EV/Tissue) in binding site abundance. Functionally significant RBPs were classified according to established

molecular ontologies[8].

Transcripts coding potential prediction

Transcripts coding potential was assessed using CPAT (v3.0.5; human model, cutoff > 0.5) and CPC2 (v0.1; default parameters). Transcripts were classified as protein-coding only when both tools yielded concordant results and the sequences contained canonical start (ATG) and stop codons (TAA, TAG, or TGA). The final genomic coordinates of open reading frames (ORFs) were obtained from CPC2.

Prediction HLA Typing

HLA class I alleles were inferred using arcasHLA (v3.9) for samples in Cohort 1, Cohort 2, and the CPTAC cohort. RNA-seq BAM files were processed through the sequential extract, genotype, partial, and merge modules of arcasHLA to generate final MHC class I haplotypes. For TCGA-PAAD samples, we utilized HLA typing results predicted by OptiType, which were downloaded from the GDC. To ensure compatibility with downstream neoantigen binding predictions using NetMHCpan, only HLA alleles supported by NetMHCpan were retained. Following this comprehensive analysis, HLA types were successfully determined for a total of 401 samples.

Identification of Mutational Neoantigens in the CPTAC and TCGA Cohorts

Annotated VCF files from Whole Exome Sequencing (WES) of the CPTAC cohort were downloaded from the GDC portal (https://portal.gdc.cancer.gov/) via the gdc-client. Gene expression data (in transcripts per million, TPM) were also retrieved from the GDC. We selected mismatch mutations and filtered for those with sequencing depth >5 and TPM >10 for mutational neoantigen prediction. SNV effect analysis was performed using snpEff.jar to derive mutated protein sequences. Using a custom script, we extracted the altered amino acid along with 11 flanking residues upstream and downstream (23 amino acids total) to ensure complete peptide context for neoantigen prediction. NetMHCpan (v4.1) was applied using the same settings and thresholds as those for TST-derived neoantigens (e.g., binding affinity cutoff, peptide length). Mutational neoantigens were further filtered to remove those present in the human reference database, excluding potential self-antigens and retaining only tumor-specific candidates.

For the TCGA cohort, we analyzed curated SNV-derived mutational neoantigens retrieved from the Tumor-Specific Neoantigen Database (TSNAdb[9]). To ensure data fidelity, we also retrieved raw somatic mutation profiles and HLA typing data from the

GDC portal and cross-validated them against TSNAdb's neoantigen predictions.

Motif analysis

For motif analysis, we employed MEME[10] Suite (v5.5.7) to perform sequence enrichment analysis on the TSS regions, comparing results against the JASPAR2022_CORE_non-redundant_v2 database. Additionally, we used WebLogo to visualize the coding TSTs of start codon sequences from CPTAC samples as well as the shared neoantigen amino acid sequences. For major HLA alleles, Gibcluster (using online default parameters) was employed to cluster neoantigens and evaluate the false discovery rate (FDR) of neoantigen identification. BLASTp was performed to identify and validate novel variant peptides and amino acid alterations using the following command 'Blastp -task blastp-short -db /uniprot_sprot -query share_len9.fa -out pep_shared.fa.txt -evalue 1000000 -max_target_seqs 100 -outfmt 6'

Single-cell RNA-seq analysis

Single-cell RNA-seq of six PDAC panties tumor and NATs were downloaded from GEO under accession GSE212966. Cellranger (v6.0.0) was used to map gene expression to the GRCh38_gencode_v29 reference genome for both tumor and NATs. Scrublet[11] (v0.2.3) was applied to identify doublet cells using the default settings. In addition to removing doublets, cells with fewer than 200 features or mitochondrial gene percentages exceeding 15% were filtered out from both tumor and NAT samples.

Seurat (v4.0.5) was employed for clustering cancer samples. The standard workflow was followed, including the commands NormalizeData, FindVariableFeatures (nfeatures = 2000), ScaleData, and RunPCA to dimensionality reduction. Using 20 dimensions, the FindNeighbors function was applied to identify neighbors, and clusters were determined with FindClusters at a resolution of 0.05. Visualization of clusters was performed using the RunUMAP function.

We used canonical markers to annotate each cluster, including epithelial cells (KRT18, KRT19, LCN2), T cells (CD3D, CD3E,CD3G), mast cells (TPSAB1, CPA3), B cells (CD79A, CD79B, MS4A1), neutrophil cells (S100A8, S100A9, LYZ), fibroblasts (COL1A2, COL3A1, COL1A1), macrophage cells (MARCO, C1QA, C1QB), and endothelial cells (CDH5, PLVAP, CLDN5), and other stromal cells (C11orf96, ADIRF, CDH19, GPM6B).

To validate the malignant nature of epithelial cell clusters, we performed copy number variation (CNV) analysis using the inferCNV package (v1.18.1). The analysis was implemented through the following steps: (1) We created an inferCNV object using the CreateInfercnvObject function, designating T cells, macrophages, B cells,

neutrophils, and mast cells as reference normal clusters; (2) CNV inference was then executed via the infercnv::run function.

Survival Analysis and Differential Gene Expression

Kaplan-Meier analysis was performed to assess the relationship between TST-derived neoantigen burden (number) and mutation neoantigen burden (number) with overall survival (OS). The survfit and Surv functions from the survival package were used, and survival curves were visualized using the ggsurvplot function. Patients in the 40th percentile of TST-derived neoantigens were classified as the low neoantigen burden group.

To examine the relationship between FOXA2 expression levels and patient overall survival (OS), we integrated FOXA2 expression data (TPM) from the CPTAC and TCGA cohorts and conducted Kaplan-Meier survival analysis to determine the optimal cutoff value. Based on survival analysis results, patients were categorized into high-expression and low-expression FOXA2 groups. Differentially expressed genes between these groups were identified using Wilcoxon tests and fold change analysis. Genes with $P < 0.01$, fold change > 2, and average expression > 1 in the high FOXA2 group were considered highly expressed in this group, while genes with $P < 0.01$, fold change < 0.5, and average expression > 1 in the low FOXA2 group were considered highly expressed in the low FOXA2 group. KEGG pathway enrichment analysis of protein-coding genes highly expressed in the low FOXA2 group was performed using the DAVID tool.

To identify neoTST associated with overall survival, specific expression thresholds were applied across different cohorts. In the CPTAC and TCGA cohorts, only junctions derived neoTSTs expressed in at least 10 samples were considered, with those exhibiting expression levels greater than 5 categorized as high expression, and the high expression group required a minimum of 5 samples. In cohort 2, junctions derived neoTSTs expressed in at least 3 samples were analyzed, with expression levels greater than 5 also classified as high expression, and the high expression group required at least 3 samples. Kaplan-Meier analysis was performed to assess the survival differences between the high and low expression groups based on optimal cutoff value.

RNA-seq Library Preparation and sequencing from Panc02 and KPC Cells

Total RNA (500 ng) was isolated from Panc02 cell line using Trizol® Reagent (Thermo Fisher, USA) followed by DNase I (NEB) treatment to eliminate genomic DNA contamination. Strand-specific libraries were constructed employing the QIAseq FastSelect-rRNA HMR (Qiagen) and KAPA RNA HyperPrep (Roche) systems. RNA fragmentation was performed at 85°C for 6 min followed by gradual cooling (75°C to 25°C over 14 min). cDNA synthesis was initiated with random hexamers, followed by

end repair (Qiagen DNA End Repair Kit), A-tailing using Klenow fragment, and adapter ligation. The resulting double-stranded DNA underwent 11 PCR amplification cycles before quality assessment using Qubit (Thermo Fisher, USA) and Qsep100 (BiOptic, China) systems. Final libraries were sequenced on DNBSEQ-T7 platform (150 bp paired-end).

Panc02 KPC and Mouse Normal Tissue RNA-Seq Analysis

Following quality control (QC) of raw RNA-seq data from Panc02, reads were aligned to the mouse reference genome GRCm38.91 (downloaded from the Ensembl database) using STAR (v2.5.3a) with parameters consistent with those described above. Transcript assemblies were generated using StringTie (v2.2.1) with the Mus_musculus.GRCm38.91 reference annotation. Gene expression levels were quantified as TPM using featureCounts[12,13] (v2.0.1).

To identify tumor-specific junctions and exons in mice, raw RNA-seq data from various normal C57BL/6 mouse tissues were obtained from the Sequence Read Archive (SRA) under accession numbers PRJNA375882, PRJNA859788, and PRJNA435886 and in house mice normal liver tissue RNA-seq, encompassing a total of 115 samples. These datasets were processed identically using STAR, StringTie, and featureCounts for alignment, transcript assembly, and expression quantification, respectively.

Identification of Panc02 and KPC Tumor-Specific Junctions and Exons

Junctions in Panc02 KPC and normal samples were identified using ASJA, while exons were annotated and quantified using the same methodology as applied to human data, involving the extraction of genomic coordinates and expression quantification based on assembled transcripts. The median expression, maximum expression, and expression frequency of junctions and exons across the normal cohort (115 samples) were calculated.

Panc02 and KPC tumor-specific junctions were defined as those with expression ≥5 CPT that met at least one of three criteria: (1) absolute novelty (completely absent in normal cohort), OR (2) low background prevalence (detected in <1% of samples in normal cohort while maintaining tumor expression ≥5× the median value of normal cohort), OR (3) robust tumor-specificity (detected in <90% of normal cohort's samples with tumor expression ≥5× the median and ≥10× the maximal value of normal cohort)

Tumor-specific exons were identified using identical thresholds, except for a minimum expression cutoff of 8. The first exon, last exon and single-exon comparison stargate were same to human

Panc02 and KPC TST-derived Neoantigen Identification

TSTs identified in Panc02 and KPC cells were merged, and their protein-coding sequences were extracted for downstream neoantigen prediction, following the same analytical pipeline used for human data. Coding potential was evaluated using CPAT with the mouse reference genome and gene annotation, applying a coding probability threshold of 0.44. All other parameters were kept consistent with the human TST analysis.

Neoantigen prediction was performed using NetMHCpan (v4.1) to identify 8-11-mer peptides with predicted binding affinity to murine MHC class I molecules H-2-Db and H-2-Kb. The selection criteria for neoantigens were identical to those applied in the human analysis. To ensure neoantigen specificity, and only peptides absent from this reference database were retained as TST-derived neoantigens.

Panc02 and KPC Mutation Detection from RNA-Seq

Three software tools were used to call candidate mutations from RNA-seq data: HaplotypeCaller[14] (v4.2.2.0), Strelka[15] (v2.9.2), and Samtools[16] (v1.6). The STAR-aligned BAM files were cleaned using Picard (v2.23.3) and GATK with the following commands in order: CleanSam, AddOrReplaceReadGroups, MarkDuplicates, SplitNCigarReads, BaseRecalibrator, and ApplyBQSR. HaplotypeCaller was employed to call mutations in the cleaned reads, with further filtering performed using VariantFiltration with the following settings: FS > 30.0, QD < 2.0, DP < 10, and AD < 5. For mutation calling with Strelka, the default command was used, and mutations were filtered based on the 'PASS' label. For Samtools, the flagstat command was used first, followed by mpileup and bcftools with default settings. Mutation variations were further filtered using the VariantFiltration function.

Panc02 and KPC Mutational Neoantigen Identification

Mutations detected by at least two independent software tools were designated as candidate mutations for neoantigen prediction. These candidates were further filtered using stringent criteria: DP >20, read coverage of the mutated base >5, and gene expression >10 TPM. To exclude germline variants, we constructed a normal tissue mutation profile using 8 non-tumor tissues (SRR20283751, SRR20283747, SRR20283735, SRR20283729, SRR20283723, SRR6781409, SRR5273669, SRR5273664). Only Panc02 mutations absent from this normal profile were retained for downstream analysis.

Subsequent neoantigen prediction was restricted to non-synonymous mutations (annotated via ANNOVAR) that passed all filtering steps. The workflow included: (1)

using snpEff.jar to derive mutated protein sequences; (2) predicting MHC binding affinity with NetMHCpan (v4.1) for 8-11 amino acid peptides (MHC class I, H-2-Db, H-2-Kb) and NetMHCIIpan (v4.1) for 14-18 amino acid peptides (MHC class II, H-2-IAb). Neoantigens with binding scores <500 were classified as weak binders ("WB") or strong binders ("SB"). Input sequences for prediction included the mutated amino acid plus 11 flanking residues (for MHC class I) or 25 flanking residues (for MHC class II) to ensure complete peptide context. Finally, we defined mutation neoantigens as those absent from the reference mouse proteome reference (to exclude self-antigens)

Representative PDAC neoTSTs

Representative PDAC neoTSTs were identified through a multi-step computational pipeline: Initial tumor specific junction excluded normal type. Retained junctions were required to exhibit elevated tumor-specific expression, defined as a maximum expression value ≥10 CPT (quantification by ASJA) in PDAC cohorts and detectable expression (>5 CPT) in at least four tumor samples. To ensure stringent normal tissue exclusion, junctions were discarded if they showed a detection frequency ≥8% in GTEx normal tissues or TCGA adjacent normals, a median expression ≥5 CPT in these cohorts, or expression in ≥5 distinct GTEx tissue types.

The remaining high-confidence junctions were ranked by their expression prevalence across PDAC tumors (CPTAC and TCGA datasets). The top candidates underwent manual validation via Integrative Genomics Viewer (IGV) to confirm read support, splicing patterns, and absence of alignment artifacts.

For single-exon neoTSTs, candidate identification was performed using the following pipeline: First, the coding sequences (CDSs) of 61 single-exon neoTSTs entirely derived from TEs were identified via bedtools intersect. Transcripts exhibiting 5' untranslated regions (5'UTRs) exceeding 500 nucleotides were excluded, yielding 18 highly-confidence candidates. These candidates were prioritized based on their expression prevalence across the CPTAC and TCGA cohorts. Finally, genomic loci supporting these neoTSTs were manually validated using the IGV, ensuring absence of mapping artifacts or read misalignment.

PANC-1 RNA-seq analysis

Total RNA from control and FOXA2-overexpressing PANC-1 cells was used for library preparation and sequenced on a DNBSEQ-T7 (150 bp PE). Raw RNA-seq reads were analyzed via NeoAPP to identify tumor-specific junctions and transcripts. Corresponding FOXA2 ChIP-seq data in PANC-1 were integrated from GSM3387447.

Peptide Synthesis and Mass Spectrometric Validation

Two candidate peptides were chemically synthesized (GenScript). Samples were analyzed by LC-MS/MS equipped with an on-line nano-electrospray ionization source. The system consisted of an Orbitrap Fusion Lumos mass spectrometer (Thermo Fisher Scientific, MA, USA) coupled to an EASY-nanoLC 1200 system. Raw MS/MS files were processed using PEAKS Studio (version 13) for peptide identification and spectral matching.

Synthesis of mRNA temple

The coding sequence for mutation neoantigens and neoTSTs (Table S5-S7) was synthesized by Dynegene company and introduced into a Takara mRNA vector (Takara Bio). Gene polyadenylation was performed using the 2× Hieff Canace® AdvanceFast PCR Master Mix (With Dye) (Yeasen, 10164ES08) and TA-F/TA-R primers, with the Takara mRNA cloning vector as the template, according to the manufacturer's protocol.

In vitro transcription (IVT)

The PCR mixture was further purified with a FastPure Gel DNA Extraction Mini Kit (Vazyme, DC301-01). In vitro transcription (IVT) was performed by adding a T7 promotor, a 120-bp poly(A) tail, a 3' UTR, followed by RNA synthesis with T7 High Yield RNA Synthesis Kit (Yeasen, 10633ES60). N1-methylpseudouridine (N1mψ)-modified mRNA was synthesized through IVT reaction. The reaction mixture was incubated at 37°C for 2 hours, then treated with DNase I (Yeasen, 10325ES80) at 37°C for 20 mins to remove template DNA. Finally, the mRNA was purified using GeneJET RNA Purification Kit (Thermo Scientific, K0731) and analyzed by 2% agarose gel electrophoresis.

Synthesis and formulation of the lipid nanoparticles

The lipid nanoparticle (LNP) formulation comprised SM-102 ionizable lipid, helper lipids (DSPC), cholesterol, and the PEG-modified lipid DMG-PEG2000, synthesized at a molar ratio of 50:10:38.5:1.5 (mol/mol). For preparation, the lipid mixture was first dissolved in ethanol, then combined with the mRNA solution (in 100 mM citrate buffer, pH 4.0) at a 3:1 volume ratio (ethanol:aqueous phases) using an INano™ L microfluidic mixer (Micro&Nano). Post-formulation, the crude LNP solution was concentrated and purified using a 100 kDa molecular weight cut-off (MWCO) ultrafiltration membrane (Millipore UFC510096). Encapsulation efficiency was determined using the Equalbit RNA HS Assay Kit (Vazyme, EQ211-01), with or without 2% Triton X-100 (Sigma, T8787) to differentiate between encapsulated and free mRNA.

Flow cytometry analysis

Splenic lymphocytes were isolated using Mouse 1× Lymphocyte Separation Medium

(DAKEWE, DKW33-R0100) according to the manufacturer's protocol. Freshly isolated cells were transfected with antigen-encoding mRNA for 16 hours using CALNP™ mRNA *in vitro* reagent (D-Nano, DN002-10). Brefeldin A (Yeasen, 50504ES08) and Monensin (Yeasen, 50501ES03) were added to the cell cultures 5 hours before harvesting the cells. The cells were collected and stained with Fixable Viability Dye eFluor™ 450 (eBioscience, 65-0863-14) to identify live cells. Then, the cells were stained with APC-conjugated anti-mouse CD3 antibody (Proteintech, APC-65077), FITC-conjugated anti-mouse CD8 antibody (Proteintech, FITC-65069) and PE/Cyanine7-conjugated anti-mouse CD4 antibody (BioLegend, 100528). Subsequently, the cells were fixed and permeabilized with an Intracellular Fixation and Permeabilization Buffer Set (eBioscience) and incubated with PE-conjugated anti-mouse IFN-γ (BioLegend, 505808) for 1 hour at 4°C. Samples were acquired using a CytoFLEX flow cytometer (Beckman).

IFN-γ ELISpot assay

The frequency of T cell activation following mRNA transfection was assessed using an IFN-γ ELISPOT assay kit (DAKEWE, 2210002). The 10 PDAC neoTST mRNAs were individually transfected into mouse splenic lymphocytes (5×10^5 cells/well) using CALNP™ mRNA *in vitro* reagent (100 ng per well), while untreated controls received no mRNA. After 24 hours of incubation, the plates were washed according to the kit instructions, followed by sequential addition of the diluted detection antibody and streptavidin-HRP. Plates were incubated at 37 °C for 1 hour. AEC substrate solution was then added to each well for color development, and the plates were left at room temperature for approximately 15-25 minutes before the reaction was stopped with deionized water.

References:

1. Quinlan AR, Hall IM. BEDTools: a flexible suite of utilities for comparing genomic features. *Bioinformatics*. 2010;26(6):841-2

2. Hao Y, Hao S, Andersen-Nissen E et al. Integrated analysis of multimodal single-cell data. *Cell*. 2021;184(13):3573-3587.e29

3. Xiang X, He Y, Zhang Z, Yang X. Interrogations of single-cell RNA splicing landscapes with SCASL define new cell identities with physiological relevance. *Nat Commun*. 2024;15(1):2164

4. Gao Y, Li J, Cheng W et al. Cross-tissue human fibroblast atlas reveals myofibroblast subtypes with

distinct roles in immune modulation. *Cancer Cell*. 2024;42(10):1764-1783.e10

5. Yu S, Li Y, Liao Z et al. Plasma extracellular vesicle long RNA profiling identifies a diagnostic signature for the detection of pancreatic ductal adenocarcinoma. *Gut*. 2020;69(3):540-550
6. Li Y, Li Y, Yu S et al. Circulating EVs long RNA-based subtyping and deconvolution enable prediction of immunogenic signatures and clinical outcome for PDAC. *Mol Ther Nucleic Acids*. 2021;26:488-501
7. Zhao J, Li Q, Hu J et al. Circular RNA landscape in extracellular vesicles from human biofluids. *Genome Med*. 2024;16(1):126
8. Van Nostrand EL, Freese P, Pratt GA et al. A large-scale binding and functional map of human RNA-binding proteins. *Nature*. 2020;583(7818):711-719
9. Wu J, Chen W, Zhou Y et al. TSNAdb v2.0: The Updated Version of Tumor-specific Neoantigen Database. *Genomics Proteomics Bioinformatics*. 2023;21(2):259-266
10. Bailey TL, Boden M, Buske FA et al. MEME SUITE: tools for motif discovery and searching. *Nucleic Acids Res*. 2009;37(Web Server issue):W202-8
11. Wolock SL, Lopez R, Klein AM. Scrublet: Computational Identification of Cell Doublets in Single-Cell Transcriptomic Data. *Cell Syst*. 2019;8(4):281-291.e9
12. Liao Y, Smyth GK, Shi W. featureCounts: an efficient general purpose program for assigning sequence reads to genomic features. *Bioinformatics*. 2014;30(7):923-30
13. Zhao Z, Chen Y, Zou X et al. Pan-cancer transcriptome analysis reveals widespread regulation through alternative tandem transcription initiation. *Sci Adv*. 2024;10(28):eadl5606
14. Brouard J, Bissonnette N. Variant Calling from RNA-seq Data Using the GATK Joint Genotyping Workflow. *Methods Mol Biol*. 2022;2493:205-233
15. Saunders CT, Wong WSW, Swamy S et al. Strelka: accurate somatic small-variant calling from sequenced tumor-normal sample pairs. *Bioinformatics*. 2012;28(14):1811-7
16. Danecek P, Bonfield JK, Liddle J et al. Twelve years of SAMtools and BCFtools. *Gigascience*. 2021;10(2)

Supplementary Figure

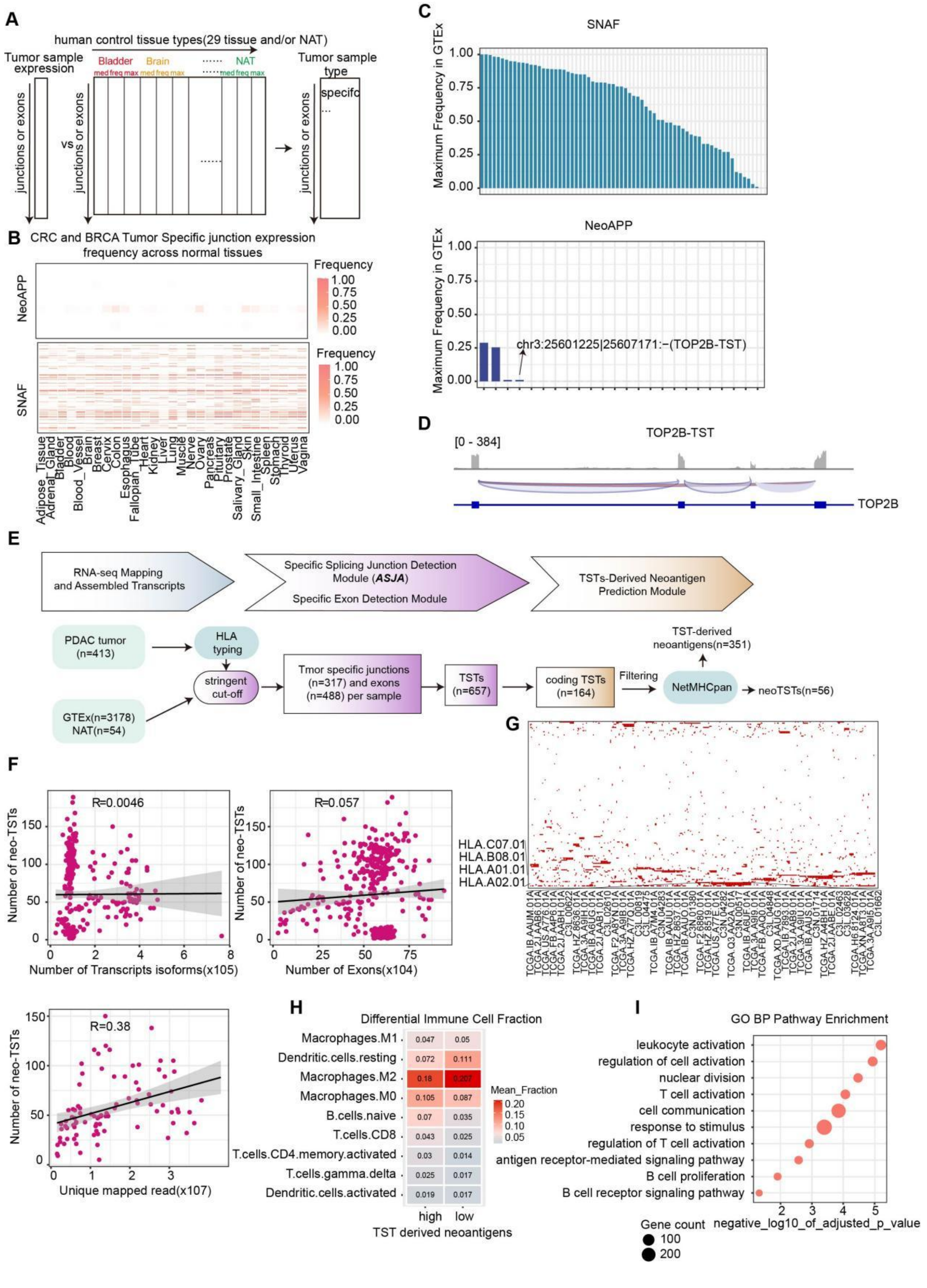

Figure S1. TST-derived neoantigen landscape in PDAC revealed by multi-cohort transcriptomic profiling. (A). The human normal tissue reference junction and exon expression matrix. (B). Heatmaps illustrate the frequency of tumor-specific splicing junctions identified by NeoAPP (up) and SNAF (bottom) in BRCA and CRC samples across 29 normal tissue types. Color gradient indicates frequency range

(0-1). (C). Frequency of neoTSTs identified by SANF and NeoAPP in GTEx across three random PDAC samples (C3N.01012 C3L.00598 and C3L.00599). (D) IGV showing the tumor specific junction identified by only NeoAPP. (E). Workflow for identifying TST-derived neoantigens: (1) RNA-seq alignment and transcript assembly; (2) systematic comparison of splicing junction and exon patterns across cohorts to detect tumor-specific transcripts (TSTs); (3) neoantigen prediction from coding TSTs. Median values were calculated from 401 eligible PDAC patients. (F). the correlation between number of neoTST and number of transcripts isofroms, number of exons and unique mapped read. (G) The heatmap displays the presence of specific HLA alleles across samples, where red indicates that a given sample carries the corresponding HLA haplotype. See detail in Supplementary table. (H) The immune cell fraction by CIBERSORT between TST derived neoantigens burden high and low in CPTAC cohort based on survival analysis. (I) The enrichment of higher expression gene in high TST derived neoantigens burden group in CPTAC cohort by gprofiler.

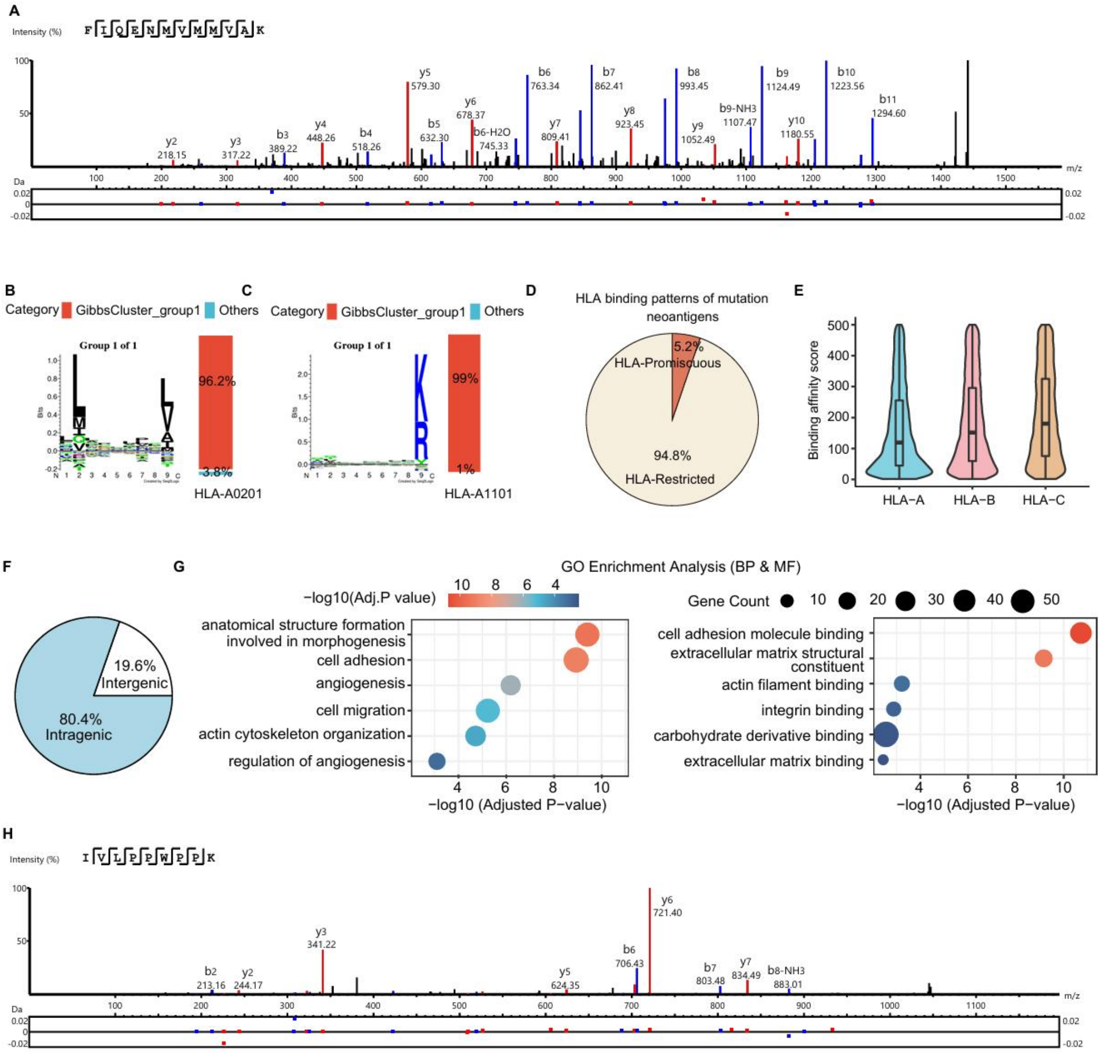

Figure S2. Proteomic validation and immunogenic primacy of TST-derived neoantigens in PDAC. (A). MS/MS spectra of the synthetic peptides FIQENMVMMVAK. (B-C). Gibbs cluster analysis for peptides that binding at HLA-A*02:01 and HLA-A*11:01. (D). Pie chart showing the proportion of mutation neoantigens binding to promiscuous HLA alleles (i.e., ≥2 HLA alleles) versus those binding to allele-specific HLAs. (E). Violin plots comparing binding affinity score of TST-derived neoantigens to HLA-A/B/C alleles. (F). Classification of neoTSTs that derived shared neoantigens. (G) GO enrichment of BP and MF for intragenic neoTSTs that derived shared neoantigens by gprofiler. (H) MS/MS spectra of the synthetic peptides IVLPPWPPK.

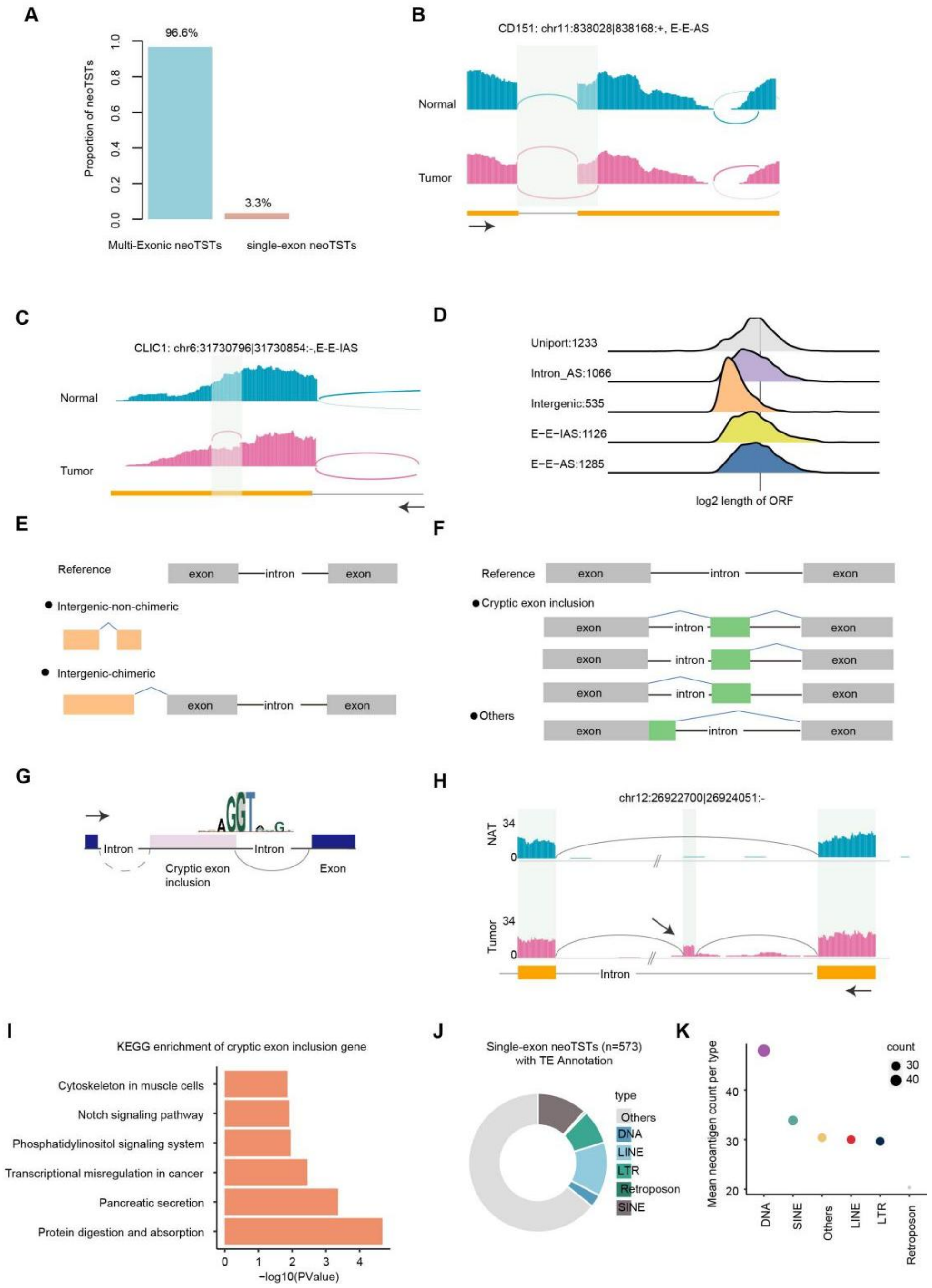

Figure S3. Non-canonical splicing and transposable element generate high-yield neoantigen expansion in PDAC. (A). 96.6% of neoTSTs are multi-exon neoTSTs. (B-C). Representative examples of E-E-AS and E-E-IAS. (D). Open reading frame (ORF) length distribution across neoTST types. (E-F). Classification of aberrant splicing subtypes in intergenic and intronic regions. (G). Sequence motifs at cryptic exon splice sites. (H). Sashimi plot of representative cryptic exon inclusion event. (I). KEGG enrichment of genes with cryptic exons. (J-K). Genomic origins of single-exonneoTSTs (J: 40% transposable elements) and their neoantigen yield.

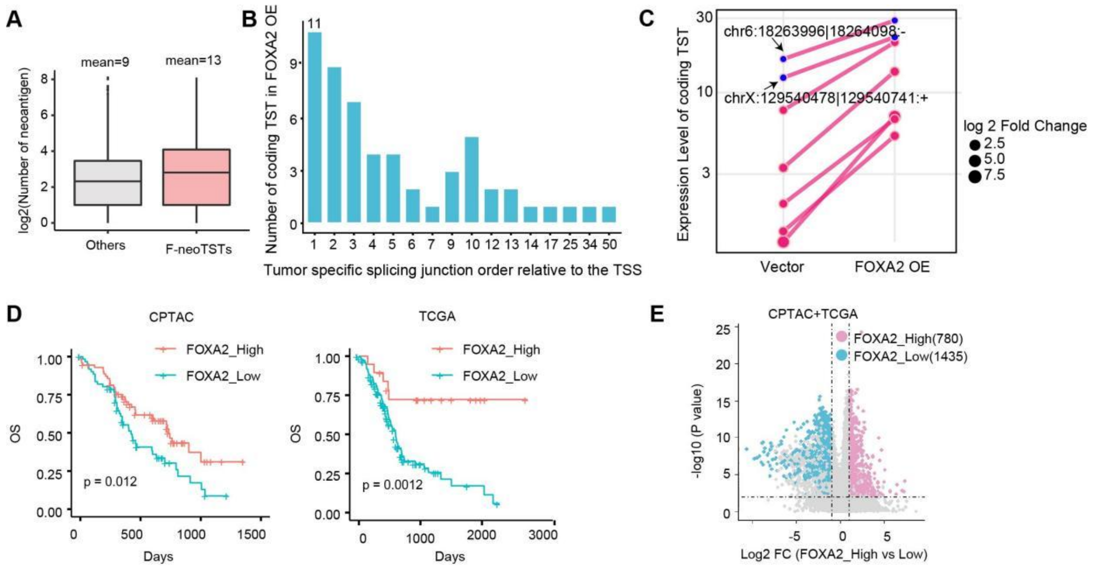

Figure S4. Alternative promoter usage as a key source of neoTSTs regulated by FOXA2. (A). Comparative histogram of TST-derived neoantigens yield between F-neoTSTs and others neoTSTs. (B). Spatial distribution of tumor-specific splicing junctions driving coding TSTs relative to the TSS. (C). Expression profiles of 7 upregulated coding TSTs derived from initial tumor-specific junction events. TSTs highlighted in the blue indicate their promoter contained FOXA2-binding sites. (D). Kaplan-Meier overall survival curves of PDAC patients stratified using the optimal FOXA2 expression threshold. The optimal cutoff point was used for stratification. (E). Volcano plot of differentially expressed genes between FOXA2-high and FOXA2-low groups.

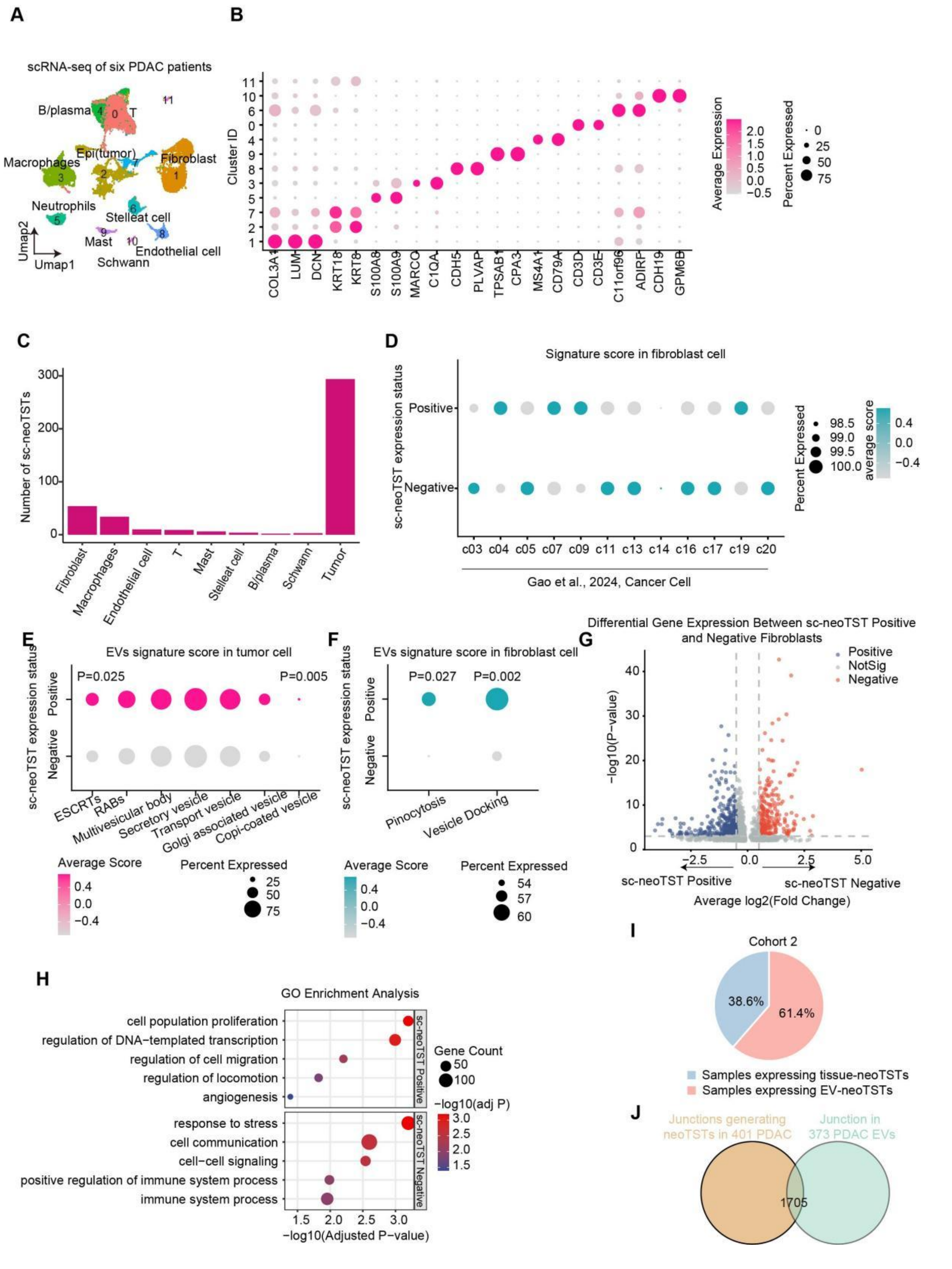

Figure S5. Distribution of neoTSTs in tumor microenvironment and extracellular vesicles . (A-B). Single-cell transcriptomic atlas of six PDAC samples with marker gene expression across cellular subpopulations. (C). The number of sc-neoTSTs among different clusters. (D). Dot plot comparing 12 fibroblast features signature scores (derived from Gao et al., Cancer Cell 2024) between sc-neoTST positive and negative fibroblast. Color intensity represents mean signature scores. (E). Dot plot evaluating EVs signature scores in tumor cells with versus without sc-neoTST expression. (F) Dot plot evaluating EVs signature scores in fibroblast cells with versus without sc-neoTST expression. (G). The DEGs between sc-neoTST positive and negative fibroblast. (H). Functional enrichment analysis based

on the identified DEGs. (I). Pie chart showing detection of neoTSTs in paired EVs across 57 samples. 61.4% of samples (n=35) showed co-detection of tissue-derived neoTSTs in EVs, while 38.6% (n=22) lacked exosomal counterparts. (J). Venn diagram comparing junction identified in PDAC EVs and tumor tissues. The intersection represents 1,705 junction conserved across both compartments.

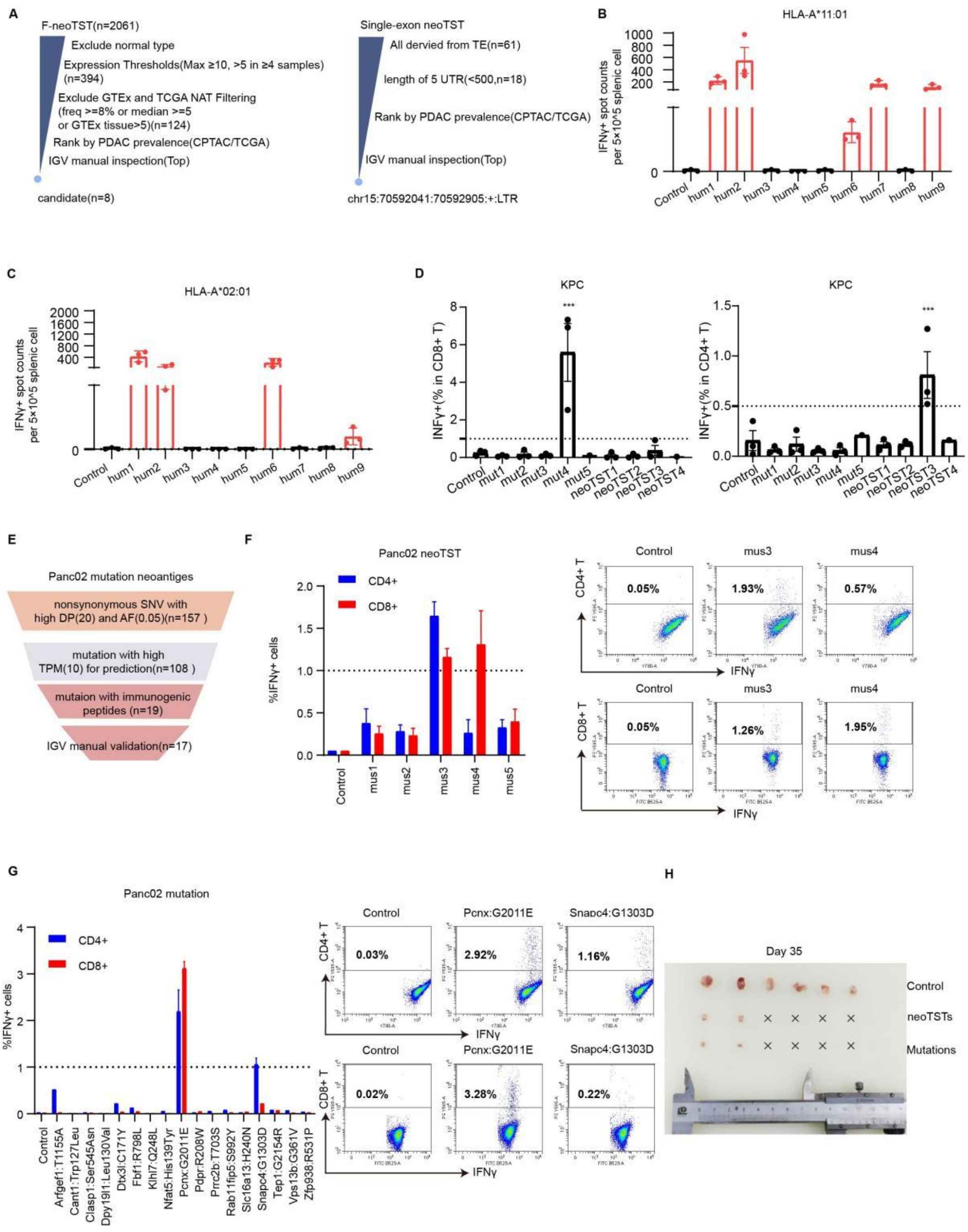

Figure S6. Functional validation of neoTSTs in transgenic and tumor-bearing murine models. (A).

Filtering criteria for F-neoTST and single-exon neoTST. (B-C). Bar graph showing the number of IFN-γ spots in response to each neoTST. Empty vector as control group. n=3. (D). Proportions of $INF\gamma^{+}CD8^{+}$ T cells and $INF\gamma^{+}CD4^{+}$ T cells induced by neoTST versus mutation-derived neoantigens. Data are presented as mean ± SEM. Statistical significance was determined by one-way ANOVA followed by Benjamini-Hochberg FDR correction for multiple comparisons. *P < 0.05, **P < 0.01, ***P < 0.001. n=3, Control: unvaccinated mice. (E). workflow for identification mutation neoantigen. (F-G). splenocytes from vaccinated Panc02 mice were isolated and were re-stimulated by neoTST (F) and mutation neoantigen (G), and $CD4^{+}$ and $CD8^{+}$ T cells were separately sorted for IFN-γ capture and assessed for reactivity by FACS. Representative plots showing $CD4^{+}$ and $CD8^{+}$ T cell activation (right). Data are presented as mean±SEM. n=1,3. (H). Tumor size measurement and visualization.

Supplementary Tables

Table S1. HLA types of each sample

Table S2. neoTST that detected in PDAC plasma EVs

Table S3. PDAC neoTSTs and their derived neoantigen for figure 6

Table S4. neoTST and mutation in KPC for validation

Table S5. Panc02 mutation neoantigen sequence

Table S6. Panc02 neoTST sequence

Table S7. PDAC neoTST sequence

Table S1. HLA types of each sample

Sample	HLA
PDAC4	HLA-C01:14,HLA-B54:01,HLA-B07:05,HLA-A02:01,HLA-A29:01,HLA-C15:05,
PDAC15	HLA-B07:05,HLA-C15:05,HLA-C01:02,HLA-A29:01,HLA-B54:01,
PDAC3	HLA-A33:03,HLA-B51:01,HLA-C14:02,HLA-B44:03,HLA-A24:02,HLA-C14:03,
PDAC28	HLA-B44:03,HLA-A24:02,HLA-C14:03,HLA-A33:03,HLA-B51:01,HLA-C14:02,
PDAC20	HLA-B40:06,HLA-C08:01,HLA-A02:01,HLA-B35:01,HLA-A24:02,
PDAC24	HLA-B40:06,HLA-C08:01,HLA-B35:01,HLA-A02:01,HLA-A24:02,HLA-C03:03,
PDAC34	HLA-C01:02,HLA-A02:07,HLA-B15:02,HLA-B46:01,HLA-C08:01,HLA-A11:01,
PDAC21	HLA-B46:01,HLA-B15:02,HLA-A02:07,HLA-C01:02,HLA-C08:01,HLA-A11:01,
PDAC30	HLA-B51:01,HLA-C14:02,HLA-B40:01,HLA-C03:04,HLA-A24:02,
PDAC33	HLA-B40:01,HLA-B51:01,HLA-A11:01,HLA-C18:02,HLA-A24:02,HLA-C03:04,
PDAC8	HLA-C03:02,HLA-B13:02,HLA-A33:03,HLA-C06:02,HLA-A30:01,HLA-B58:01,
PDAC16	HLA-C06:02,HLA-A30:01,HLA-B58:01,HLA-A33:03,HLA-B13:02,HLA-C03:02,
PDAC2	HLA-B46:01,HLA-C01:02,HLA-B15:18,HLA-A02:07,HLA-C08:01,HLA-A11:01,
PDAC9	HLA-B46:01,HLA-C01:02,HLA-A02:07,HLA-B15:18,HLA-C08:01,HLA-A11:01,
PDAC11	HLA-A02:07,HLA-A24:02,HLA-C01:02,HLA-B46:01,HLA-C14:02,HLA-B51:01,
PDAC31	HLA-C14:02,HLA-B51:01,HLA-B46:01,HLA-C01:02,HLA-A02:07,HLA-A24:02,
PDAC18	HLA-B13:01,HLA-C04:01,HLA-C03:04,HLA-A30:02,HLA-B53:01,
PDAC7	HLA-A11:01,HLA-B13:01,HLA-C04:01,HLA-C03:04,HLA-A30:02,HLA-B53:01,
PDAC5	HLA-C01:02,HLA-A24:02,HLA-B46:01,HLA-B54:01,HLA-A11:01,
PDAC10	HLA-B46:01,HLA-B54:01,HLA-C01:02,HLA-A24:02,HLA-A11:01,
PDAC23	HLA-C03:04,HLA-B37:01,HLA-A11:01,HLA-A01:01,HLA-B40:01,HLA-C06:02,
PDAC25	HLA-B40:01,HLA-B37:01,HLA-C03:04,HLA-C06:02,HLA-A01:01,HLA-A11:01,
PDAC22	HLA-B40:01,HLA-B58:01,HLA-A33:03,HLA-C03:02,
PDAC14	HLA-C03:02,HLA-A33:03,HLA-B40:01,HLA-B58:01,
PDAC29	HLA-C14:03,HLA-B44:03,HLA-C07:06,HLA-B13:01,HLA-A11:01,HLA-A33:03,
PDAC19	HLA-B44:03,HLA-C14:03,HLA-A33:03,HLA-B44:02,HLA-C07:06,
PDAC17	HLA-B44:03,HLA-C14:03,HLA-A02:01,HLA-C15:02,HLA-A33:03,HLA-B40:01,
PDAC26	HLA-A33:03,HLA-C15:02,HLA-B40:01,HLA-B44:03,HLA-A02:01,HLA-C14:03,
PDAC12	HLA-B45:01,HLA-C03:02,HLA-B58:01,HLA-C06:02,HLA-A23:01,HLA-A33:03,
PDAC32	HLA-C06:02,HLA-B58:01,HLA-A23:01,HLA-A33:03,HLA-B45:01,HLA-C03:02,
PDAC6	HLA-A02:01,HLA-C15:02,HLA-A31:01,HLA-B40:01,HLA-B51:01,
PDAC13	HLA-A31:01,HLA-C15:02,HLA-B51:01,HLA-B40:01,HLA-A02:01,
PDAC1	HLA-B40:01,HLA-C07:02,HLA-B55:02,HLA-A31:01,HLA-A02:01,HLA-C01:02,
PDAC27	HLA-C07:02,HLA-B40:01,HLA-A31:01,HLA-B55:02,HLA-A02:01,HLA-C01:02,
T12.10452F	HLA-A69:01,HLA-C12:08,HLA-C15:02,HLA-B52:01,HLA-B40:01,HLA-A02:01,
T12.12067E	HLA-B39:01,HLA-C07:02,HLA-C12:02,HLA-B27:47,
T12.1518E	HLA-B35:01,HLA-C03:03,HLA-C07:02,HLA-A02:06,HLA-B40:01,HLA-A11:01,
T12.21368B	HLA-A02:01,HLA-B40:06,HLA-B35:01,
T12.22407J	HLA-B44:02,HLA-B13:25,
T12.22572G	HLA-B46:01,HLA-B27:04,HLA-C12:02,HLA-A02:01,HLA-A11:01,HLA-C01:02,
T12.24151B	HLA-C03:04,HLA-B35:01,HLA-A11:75,HLA-C08:01,HLA-B15:02,HLA-A02:01,
T12.24796B	HLA-A26:01,HLA-C04:01,HLA-B15:01,HLA-A11:01,HLA-B46:24,HLA-C01:06,
T12.25712A	HLA-C15:04,HLA-C03:04,HLA-A11:01,HLA-A11:05,HLA-B51:01,HLA-B13:01,
T12.27625O	HLA-B39:34,HLA-C01:02,HLA-C07:29,HLA-A24:02,HLA-B55:02,
T12.30433D	HLA-B13:02,HLA-C03:02,HLA-B58:01,HLA-A31:01,HLA-A30:01,HLA-C06:02,
T12.31032F	HLA-C08:01,HLA-C03:04,HLA-B15:11,HLA-A02:01,HLA-B58:01,HLA-A33:03,
T12.31866G	HLA-C01:02,HLA-A24:02,HLA-C14:02,HLA-B51:01,HLA-B46:01,
T12.31963B	HLA-C01:02,HLA-B15:01,HLA-C04:01,HLA-B51:01,HLA-A11:01,HLA-A02:01,
T12.32169F	HLA-A69:01,HLA-B40:02,HLA-B56:01,HLA-C03:04,HLA-A30:01,HLA-C07:02,

T12.33421E	HLA-B40:06,HLA-A02:07,HLA-B46:01,HLA-A24:02,HLA-C01:02,HLA-C08:01,
T12.33450D	HLA-C01:02,HLA-A11:02,HLA-A02:01,HLA-B58:01,HLA-C03:02,HLA-B46:01,
T12.33555B	HLA-A02:01,HLA-B13:02,HLA-C03:04,HLA-B37:02,
T12.36812B	HLA-C06:02,HLA-B54:01,HLA-B57:01,HLA-A26:01,HLA-A02:01,HLA-C01:30,
T12.4182F	HLA-B13:02,HLA-A30:01,HLA-B15:01,HLA-C06:02,HLA-A02:01,HLA-C01:02,
T12.4329H	HLA-A02:01,HLA-A33:03,HLA-B58:01,HLA-B15:02,HLA-C08:01,HLA-C03:04,
T12.4451G	HLA-B52:01,HLA-A02:06,HLA-B51:01,HLA-C12:02,HLA-C14:02,
T12.4827H	HLA-B13:01,HLA-B13:02,HLA-C06:02,HLA-A11:01,HLA-C03:04,HLA-A30:01,
T12.5110B	HLA-B51:01,HLA-A02:07,HLA-C14:02,HLA-B55:02,
T12.5225E	HLA-A02:01,HLA-B51:01,HLA-B46:01,HLA-A24:02,HLA-C01:02,HLA-C14:02,
T12.7602D	HLA-B13:02,HLA-C08:01,HLA-A11:02,HLA-A24:02,HLA-B15:02,HLA-C06:02,
T12.7790L	HLA-A01:01,HLA-A24:02,HLA-C01:02,HLA-B54:01,HLA-B15:01,
T12.9038D	HLA-B51:01,HLA-A02:01,HLA-B46:01,HLA-A24:07,
T13.11354C	HLA-B52:01,HLA-A02:01,HLA-B53:01,HLA-A24:02,HLA-C08:01,HLA-C14:02,
T13.11544A	HLA-A11:01,HLA-C07:02,HLA-B39:01,HLA-C03:04,HLA-A02:01,HLA-B40:06,
T13.11550B	HLA-C01:30,HLA-B40:01,HLA-B55:01,HLA-A11:01,HLA-A24:02,HLA-C07:02,
T13.12038B	HLA-B40:02,HLA-A02:07,HLA-C04:01,HLA-B15:27,HLA-C08:01,HLA-A24:02,
T13.13227D	HLA-B51:01,HLA-B54:01,HLA-C14:02,HLA-C01:02,
T13.1334A	HLA-A02:03,HLA-C03:04,HLA-C06:02,HLA-B13:01,HLA-B58:01,HLA-A33:03,
T13.13801A	HLA-A29:01,HLA-B07:05,HLA-B58:01,HLA-A33:03,HLA-C03:02,HLA-C15:02,
T13.1523B	HLA-B40:06,HLA-C08:22,HLA-A24:02,HLA-A11:01,HLA-B15:02,HLA-C08:01,
T13.15336B	HLA-C08:01,HLA-B15:02,HLA-B13:02,HLA-A01:01,HLA-A02:06,
T13.16451A	HLA-B35:01,HLA-A30:01,HLA-C06:02,HLA-A24:02,HLA-B13:02,HLA-C03:02,
T13.16558B	HLA-B15:11,HLA-A11:01,HLA-C03:04,HLA-C04:01,HLA-A02:01,
T13.18374B	HLA-B40:01,HLA-A24:02,HLA-A11:01,HLA-C07:02,
T13.19137D	HLA-C07:02,HLA-C12:02,HLA-A11:01,HLA-B39:09,HLA-A02:03,HLA-B52:01,
T13.19948D	HLA-C03:02,HLA-A33:03,HLA-B58:01,HLA-B40:01,HLA-A02:07,HLA-C07:02,
T13.20105D	HLA-B15:27,HLA-A02:07,HLA-C04:01,HLA-B40:01,HLA-C03:04,HLA-A02:05,
T13.20667B	HLA-C03:02,HLA-A24:142,HLA-B51:01,HLA-B27:04,HLA-C12:02,HLA-A24:02,
T13.21819C	HLA-C07:172,HLA-B46:01,HLA-B38:02,HLA-C01:02,HLA-A11:01,HLA-A24:02,
T13.24931C	HLA-B51:01,HLA-B13:02,HLA-C06:02,HLA-A30:01,HLA-C14:02,HLA-A11:02,
T13.2594B	HLA-B15:01,HLA-A02:03,HLA-A24:02,HLA-C03:02,HLA-C04:01,HLA-B58:01,
T13.35318B	HLA-C07:02,HLA-C01:02,HLA-B15:12,HLA-A11:01,HLA-B40:01,
T13.3746E	HLA-C01:02,HLA-A02:01,HLA-C03:04,HLA-B13:01,HLA-A02:07,HLA-B46:01,
T13.4566F	HLA-B15:01,HLA-C01:02,HLA-B44:03,HLA-C14:03,HLA-A02:07,HLA-A33:03,
T13.487B	HLA-B40:01,HLA-A24:20,HLA-B40:172,HLA-C07:02,HLA-C04:01,
T13.4917F	HLA-A02:07,HLA-B46:01,HLA-C03:03,HLA-C01:02,HLA-A24:02,HLA-B15:01,
T13.5445B	HLA-C14:02,HLA-B15:01,HLA-B51:01,HLA-A66:17,HLA-A02:01,HLA-C03:03,
T13.7268B	HLA-A02:79,HLA-B58:01,HLA-C01:08,HLA-C03:46,HLA-B54:01,HLA-A24:02,
T13.7825B	HLA-B13:12,HLA-C04:01,HLA-A02:01,HLA-C07:02,HLA-A24:02,HLA-B40:01,
T13.7867D	HLA-B13:02,HLA-A11:01,HLA-A30:01,HLA-B15:01,
T13.79A	HLA-C07:02,HLA-C01:02,HLA-A02:01,HLA-B67:01,HLA-B46:01,
C3L.00017	HLA-C05:01,HLA-B44:02,HLA-B37:01,HLA-C06:02,HLA-A01:01,HLA-A29:02
C3L.00102	HLA-A68:01,HLA-A02:01,HLA-B44:02,HLA-C05:01
C3L.00189	HLA-C02:02,HLA-B51:01,HLA-C15:02,HLA-A02:01,HLA-B27:05,HLA-A24:02
C3L.00401	HLA-C07:02,HLA-A26:01,HLA-B45:01,HLA-B07:02,HLA-A03:01,HLA-C06:02
C3L.00598	HLA-A23:01,HLA-B55:01,HLA-C03:03,HLA-A11:01,HLA-B44:03,HLA-C04:01
C3L.00599	HLA-B07:02,HLA-C04:01,HLA-C07:02,HLA-A02:01,HLA-A24:02,HLA-B44:03
C3L.00625	HLA-B18:01,HLA-A11:01,HLA-B35:01,HLA-A24:02,HLA-C07:01,HLA-C04:01
C3L.00881	HLA-A32:01,HLA-C03:04,HLA-B40:01,HLA-A24:02
C3L.01031	HLA-B15:01,HLA-A03:01,HLA-C04:01,HLA-C03:03,HLA-B35:01,HLA-A24:02

C3L.01036	HLA-B38:01,HLA-C12:03,HLA-B44:02,HLA-A03:01,HLA-C05:01,HLA-A26:01
C3L.01037	HLA-C06:02,HLA-B44:02,HLA-C05:01,HLA-A02:01,HLA-B13:02
C3L.01051	HLA-C04:01,HLA-C07:01,HLA-A02:01,HLA-B18:01,HLA-A11:01,HLA-B35:01
C3L.01052	HLA-C12:05,HLA-C15:13,HLA-A03:01,HLA-B51:01,HLA-A24:02,HLA-B18:01
C3L.01054	HLA-B35:01,HLA-A01:01,HLA-C06:02,HLA-A03:01,HLA-B35:02,HLA-C04:01
C3L.01124	HLA-B07:02,HLA-A02:01,HLA-C07:02,HLA-A01:01
C3L.01328	HLA-C03:04,HLA-B35:03,HLA-C01:02,HLA-A11:01,HLA-A02:01,HLA-B27:05
C3L.01598	HLA-B37:01,HLA-C06:02,HLA-C03:04,HLA-B15:01,HLA-A11:01,HLA-A02:01
C3L.01637	HLA-A03:01,HLA-B07:02,HLA-C07:01,HLA-B08:01,HLA-C07:02,HLA-A01:01
C3L.01662	HLA-C07:01,HLA-C12:03,HLA-A25:01,HLA-A01:01,HLA-B08:01,HLA-B18:01
C3L.01687	HLA-A11:01,HLA-A33:03,HLA-B44:03,HLA-C01:03,HLA-B46:01,HLA-C14:03
C3L.01971	HLA-A01:01,HLA-B40:01,HLA-B40:02,HLA-A68:01,HLA-C02:02,HLA-C03:04
C3L.02109	HLA-B14:02,HLA-B51:01,HLA-C08:02,HLA-A02:01,HLA-C14:02
C3L.02112	HLA-B07:02,HLA-A03:01,HLA-C02:02,HLA-B51:01,HLA-C07:02,HLA-A11:01
C3L.02115	HLA-B44:02,HLA-C07:01,HLA-B18:01,HLA-A02:01,HLA-C07:04
C3L.02116	HLA-A24:02,HLA-B40:01,HLA-C07:02,HLA-B13:01,HLA-C03:04
C3L.02118	HLA-A02:07,HLA-A33:03,HLA-C03:02,HLA-B13:01,HLA-C03:04,HLA-B58:01
C3L.02463	HLA-B08:01,HLA-B44:03,HLA-A01:01,HLA-C16:01,HLA-A29:02,HLA-C07:01
C3L.02606	HLA-B27:05,HLA-A24:02,HLA-C01:02,HLA-A01:01,HLA-B35:01
C3L.02610	HLA-C01:02,HLA-B08:01,HLA-B27:05,HLA-A02:01,HLA-C07:01,HLA-A03:01
C3L.02613	HLA-C01:02,HLA-A24:02,HLA-A02:07,HLA-C03:04,HLA-B13:01,HLA-B46:01
C3L.02701	HLA-B18:01,HLA-A02:01,HLA-C07:01
C3L.02897	HLA-C12:03,HLA-A32:01,HLA-B38:01,HLA-C02:02,HLA-B40:02,HLA-A26:01
C3L.02899	HLA-C05:01,HLA-C07:02,HLA-A02:01,HLA-B07:04,HLA-B44:02
C3L.03356	HLA-C05:01,HLA-A24:02,HLA-C08:02,HLA-B14:02,HLA-B44:02,HLA-A68:02
C3L.03388	HLA-B48:01,HLA-B18:01,HLA-A02:01,HLA-C07:01,HLA-A02:06,HLA-C08:01
C3L.03394	HLA-B18:01,HLA-A02:01,HLA-B55:01,HLA-A24:02,HLA-C01:02,HLA-C03:03
C3L.03395	HLA-A24:02,HLA-A02:01,HLA-C04:01,HLA-B57:01,HLA-B35:02,HLA-C06:02
C3L.03628	HLA-A29:02,HLA-C07:01,HLA-A01:01,HLA-B44:03,HLA-C16:01,HLA-B08:01
C3L.03630	HLA-B44:02,HLA-B07:02,HLA-A03:01,HLA-C06:02,HLA-A30:01,HLA-C07:02
C3L.03635	HLA-B44:02,HLA-C05:01,HLA-A02:01
C3L.04027	HLA-A02:01,HLA-B35:01,HLA-A03:01,HLA-B51:01,HLA-C15:02,HLA-C04:01
C3L.04072	HLA-C05:01,HLA-B18:01,HLA-B53:01,HLA-A02:02,HLA-A29:02,HLA-C04:01
C3L.04473	HLA-C06:02,HLA-C17:01,HLA-A23:01,HLA-A24:02,HLA-B50:01,HLA-B41:01
C3L.04479	HLA-B37:01,HLA-B14:02,HLA-C06:02,HLA-A02:01,HLA-C08:02,HLA-A01:01
C3L.04495	HLA-B44:02,HLA-C06:02,HLA-C05:01,HLA-A02:01,HLA-B13:02
C3N.00198	HLA-B07:02,HLA-A03:01,HLA-C04:01,HLA-C07:02,HLA-A02:01,HLA-B44:03
C3N.00249	HLA-B35:03,HLA-A01:01,HLA-A03:01,HLA-C04:01,HLA-C12:03
C3N.00302	HLA-C01:02,HLA-B27:05,HLA-C07:02,HLA-A26:01,HLA-B07:02,HLA-A03:01
C3N.00436	HLA-B51:01,HLA-C06:02,HLA-B50:01,HLA-C01:02,HLA-A02:01
C3N.00512	HLA-B18:01,HLA-B35:03,HLA-A68:01,HLA-A25:01,HLA-C04:01,HLA-C12:03
C3N.00517	HLA-A01:01,HLA-B08:01,HLA-A02:01,HLA-B57:01,HLA-C07:01,HLA-C06:02
C3N.00709	HLA-A02:01,HLA-C03:03,HLA-B15:01,HLA-C03:04
C3N.00957	HLA-A03:01,HLA-C03:04,HLA-C04:01,HLA-B51:02,HLA-A24:02,HLA-B40:01
C3N.01012	HLA-A03:01,HLA-B07:02,HLA-C07:01,HLA-B49:01,HLA-C07:02,HLA-A02:01
C3N.01166	HLA-A11:01,HLA-A01:01,HLA-B08:01,HLA-B13:02,HLA-C07:01,HLA-C04:01
C3N.01167	HLA-A68:01,HLA-B35:03,HLA-A26:01,HLA-C12:03,HLA-C04:01,HLA-B38:01
C3N.01168	HLA-C01:02,HLA-A26:01,HLA-B27:05,HLA-A02:01,HLA-B57:01,HLA-C06:02
C3N.01382	HLA-B57:01,HLA-C06:02,HLA-A03:01,HLA-B07:02,HLA-C07:02,HLA-A02:01
C3N.01502	HLA-C08:02,HLA-A33:01,HLA-A68:02,HLA-B14:02
C3N.01715	HLA-C03:03,HLA-B35:01,HLA-A01:01,HLA-B15:01,HLA-A32:01,HLA-C04:01

C3N.01719	HLA-B27:05,HLA-A02:01,HLA-C02:02,HLA-C03:04
C3N.01997	HLA-A02:06,HLA-B15:01,HLA-A24:02,HLA-C01:02,HLA-B35:01,HLA-C03:03
C3N.02010	HLA-B41:01,HLA-C17:01,HLA-A02:02,HLA-A68:02,HLA-C07:18,HLA-B58:01
C3N.02295	HLA-C06:02,HLA-B49:01,HLA-B57:01,HLA-C07:01,HLA-A02:01,HLA-A23:01
C3N.02579	HLA-C01:02,HLA-A24:10,HLA-A11:01,HLA-C07:04,HLA-B18:02,HLA-B15:01
C3N.02585	HLA-B51:01,HLA-A31:01,HLA-C14:02,HLA-C03:03,HLA-B35:01,HLA-A24:02
C3N.02589	HLA-A32:01,HLA-B15:01,HLA-C03:04,HLA-B35:01,HLA-C03:03,HLA-A02:01
C3N.02592	HLA-C03:04,HLA-C04:01,HLA-A29:01,HLA-A25:01,HLA-B40:01,HLA-B35:01
C3N.02768	HLA-C07:02,HLA-B56:01,HLA-B35:01,HLA-C03:03,HLA-A11:01,HLA-A33:03
C3N.02940	HLA-B51:01,HLA-C15:02,HLA-C07:02,HLA-A02:01,HLA-B40:01,HLA-A11:01
C3N.02998	HLA-C03:04,HLA-B15:01,HLA-C12:03,HLA-B38:01,HLA-A26:01,HLA-A02:01
C3N.03000	HLA-A03:01,HLA-B38:01,HLA-C12:03,HLA-A26:01
C3N.03007	HLA-A32:01,HLA-C16:02,HLA-B15:18,HLA-B07:02,HLA-A24:02,HLA-C07:04
C3N.03039	HLA-A23:01,HLA-B18:01,HLA-A24:02,HLA-C07:01,HLA-C12:03,HLA-B49:01
C3N.03173	HLA-C14:02,HLA-A02:01,HLA-C01:02,HLA-B51:01
C3N.03190	HLA-B40:01,HLA-A24:02,HLA-A02:06,HLA-C04:01,HLA-B15:27,HLA-C15:02
C3N.03211	HLA-C15:05,HLA-A11:02,HLA-A11:01,HLA-C12:02,HLA-B07:05,HLA-B27:04
C3N.03426	HLA-A02:01,HLA-B39:01,HLA-C07:02,HLA-A01:01,HLA-C03:03,HLA-B15:01
C3N.03428	HLA-B51:01,HLA-C07:01,HLA-B08:01,HLA-A11:01,HLA-C03:03
C3N.03439	HLA-A25:01,HLA-A01:01,HLA-B08:01,HLA-B18:01,HLA-C07:01,HLA-C12:03
C3N.03440	HLA-A02:01,HLA-B08:01,HLA-A01:01,HLA-B51:01,HLA-C15:02,HLA-C07:01
C3N.03665	HLA-C07:01,HLA-C04:01,HLA-B39:24,HLA-B15:01,HLA-A02:01
C3N.03666	HLA-C08:02,HLA-A26:01,HLA-C12:03,HLA-B38:01,HLA-A68:02,HLA-B14:02
C3N.03670	HLA-B55:01,HLA-A23:01,HLA-B50:01,HLA-C03:03,HLA-A11:01,HLA-C06:02
C3N.03780	HLA-B44:02,HLA-A02:01,HLA-B55:01,HLA-C05:01,HLA-A26:01,HLA-C03:03
C3N.03839	HLA-B55:02,HLA-B15:07,HLA-A11:01,HLA-C03:03,HLA-A26:01
C3N.03840	HLA-A30:01,HLA-A11:01,HLA-B15:02,HLA-C04:01,HLA-B15:01,HLA-C08:01
C3N.03884	HLA-C12:02,HLA-C08:01,HLA-A30:01,HLA-B27:04,HLA-B48:01,HLA-A02:01
C3N.04284	HLA-A68:01,HLA-C15:02,HLA-B51:01,HLA-B44:02,HLA-A01:01,HLA-C07:04
C3L.00277	HLA-A68:01,HLA-C05:01,HLA-C07:04,HLA-B27:05,HLA-A03:01,HLA-B44:02
C3L.00589	HLA-B13:02,HLA-A30:01,HLA-B44:05,HLA-C06:02,HLA-C02:02,HLA-A01:01
C3L.00622	HLA-A01:01,HLA-B40:01,HLA-C03:04,HLA-B08:01,HLA-A31:01,HLA-C07:01
C3L.00640	HLA-A02:01,HLA-C05:01,HLA-B44:02,HLA-C08:02,HLA-B14:01
C3L.00819	HLA-C07:01,HLA-B49:01,HLA-B08:01,HLA-A03:02,HLA-A01:01
C3L.00928	HLA-C06:02,HLA-C03:04,HLA-B40:01,HLA-A30:01,HLA-B13:02,HLA-A02:01
C3L.01053	HLA-B13:02,HLA-C06:02,HLA-C02:02,HLA-B07:02,HLA-A24:02,HLA-A32:01
C3L.01453	HLA-B38:01,HLA-A26:01,HLA-A24:02,HLA-C04:01,HLA-B35:02,HLA-C12:03
C3L.01689	HLA-C07:02,HLA-A31:01,HLA-A32:01,HLA-B51:01,HLA-B07:02,HLA-C15:02
C3L.01703	HLA-B41:01,HLA-A32:01,HLA-A02:01,HLA-C12:03,HLA-C17:01,HLA-B18:01
C3L.02604	HLA-B07:02,HLA-C03:04,HLA-B40:01,HLA-C07:02,HLA-A02:01
C3L.02809	HLA-B27:02,HLA-B41:01,HLA-A24:02,HLA-C17:01,HLA-C02:02
C3L.02890	HLA-C07:18,HLA-A03:01,HLA-B58:01,HLA-A01:01,HLA-B37:01,HLA-C06:02
C3L.03123	HLA-A68:01,HLA-A03:01,HLA-B07:02,HLA-C07:02,HLA-C07:04,HLA-B44:02
C3L.03632	HLA-B18:01,HLA-C06:02,HLA-C07:01,HLA-A02:01,HLA-A24:02,HLA-B57:01
C3L.03639	HLA-B40:01,HLA-C03:04,HLA-A02:06,HLA-C08:03,HLA-B48:01,HLA-A24:02
C3L.03743	HLA-A02:01,HLA-A29:02,HLA-C06:02,HLA-C03:04,HLA-B40:01,HLA-B45:01
C3L.04080	HLA-C12:03,HLA-C02:02,HLA-B44:02,HLA-B38:01,HLA-A26:01,HLA-A24:02
C3L.04475	HLA-B49:01,HLA-A24:02,HLA-A31:01,HLA-C07:01,HLA-B08:01
C3L.04848	HLA-C05:01,HLA-C07:01,HLA-B44:02,HLA-A23:01,HLA-B08:01,HLA-A01:01
C3L.04853	HLA-B57:01,HLA-C07:02,HLA-A02:01,HLA-A03:01,HLA-B07:02,HLA-C06:02
C3N.00303	HLA-B40:01,HLA-C03:04,HLA-C06:02,HLA-A01:01,HLA-A02:01,HLA-B57:01

C3N.00511	HLA-C05:01,HLA-A02:01,HLA-B41:02,HLA-A66:01,HLA-B44:02,HLA-C17:01
C3N.00513	HLA-A03:01,HLA-B07:02,HLA-C04:01,HLA-A23:01,HLA-C07:02,HLA-B44:03
C3N.00514	HLA-A01:01,HLA-C04:01,HLA-B35:03,HLA-B08:01,HLA-A11:01,HLA-C07:01
C3N.00516	HLA-A02:01,HLA-B15:01,HLA-C07:02,HLA-C03:04,HLA-B07:02,HLA-A03:01
C3N.00518	HLA-B39:01,HLA-B27:05,HLA-A02:01,HLA-C12:03,HLA-C02:02
C3N.01011	HLA-B40:01,HLA-C03:04,HLA-B44:02,HLA-A02:01,HLA-A31:01,HLA-C05:01
C3N.01165	HLA-A01:01,HLA-B13:02,HLA-C06:02,HLA-B44:02,HLA-C05:01,HLA-A02:01
C3N.01375	HLA-B44:03,HLA-B40:02,HLA-A23:01,HLA-C02:02,HLA-A68:01,HLA-C04:01
C3N.01380	HLA-B08:01,HLA-B27:05,HLA-A02:01,HLA-C07:01,HLA-A01:01,HLA-C02:02
C3N.01381	HLA-C03:04,HLA-B40:01,HLA-C02:02,HLA-A03:01,HLA-A02:01,HLA-B27:05
C3N.01383	HLA-C17:01,HLA-C03:04,HLA-B41:01,HLA-B15:01,HLA-A02:01,HLA-A32:01
C3N.01388	HLA-B44:02,HLA-A02:01,HLA-A11:12,HLA-C07:01,HLA-C05:01,HLA-B18:01
C3N.01714	HLA-A01:01,HLA-C07:01,HLA-A02:01,HLA-B44:03,HLA-C16:02,HLA-B08:01
C3N.01716	HLA-B37:01,HLA-C06:02,HLA-B44:02,HLA-A02:01,HLA-C05:01
C3N.01998	HLA-C01:02,HLA-B54:01,HLA-C03:04,HLA-A02:06,HLA-A24:02,HLA-B40:02
C3N.02573	HLA-B39:01,HLA-A24:02,HLA-C07:02
C3N.02944	HLA-A02:01,HLA-A11:02,HLA-C12:02,HLA-B27:04,HLA-B15:11,HLA-C03:03
C3N.02971	HLA-B07:02,HLA-A01:01,HLA-A03:01,HLA-C12:03,HLA-B38:01,HLA-C07:02
C3N.03006	HLA-B18:01,HLA-A01:01,HLA-C07:01,HLA-A02:01,HLA-C07:02,HLA-B39:06
C3N.03061	HLA-A24:02,HLA-B27:05,HLA-C02:02,HLA-C04:01,HLA-B35:02,HLA-A03:01
C3N.03069	HLA-C07:02,HLA-A11:01,HLA-A24:02,HLA-B39:01,HLA-C01:02,HLA-B54:01
C3N.03086	HLA-C01:02,HLA-A02:07,HLA-B07:02,HLA-B46:01,HLA-C07:02,HLA-A02:01
C3N.03430	HLA-B38:01,HLA-A26:01,HLA-B14:02,HLA-A33:01,HLA-C12:03,HLA-C08:02
C3N.03754	HLA-A26:01,HLA-A02:01,HLA-B51:01,HLA-C04:01,HLA-B50:01,HLA-C06:02
C3N.03853	HLA-A02:03,HLA-B46:01,HLA-C07:02,HLA-A02:06,HLA-B40:01,HLA-C01:02
C3N.04119	HLA-C03:02,HLA-B27:04,HLA-C12:02,HLA-A24:02,HLA-B58:01
C3N.04126	HLA-C06:02,HLA-A30:01,HLA-B13:02,HLA-B07:02,HLA-A24:02,HLA-C07:02
C3N.04282	HLA-C07:01,HLA-B08:01,HLA-B50:01,HLA-C06:02,HLA-A02:05,HLA-A01:01
C3N.04283	HLA-B08:01,HLA-C07:01,HLA-B41:02,HLA-A66:01,HLA-A01:01,HLA-C17:01
TCGA.IB.7891.01A	HLA-A02:01,HLA-A29:02,HLA-B38:01,HLA-B18:01,HLA-C16:01,HLA-C12:03
TCGA.IB.AAUU.01A	HLA-A02:01,HLA-A01:01,HLA-B08:01,HLA-B18:04,HLA-C07:01,HLA-C12:03
TCGA.FB.AAQ2.01A	HLA-A66:01,HLA-A03:01,HLA-B35:01,HLA-B41:02,HLA-C17:01,HLA-C04:01
TCGA.3A.A9IS.01A	HLA-A24:02,HLA-A03:01,HLA-B07:02,HLA-B07:02,HLA-C07:02,HLA-C07:02
TCGA.YY.A8LH.01A	HLA-A01:01,HLA-A03:01,HLA-B50:01,HLA-B35:01,HLA-C06:02,HLA-C04:01
TCGA.S4.A8RO.01A	HLA-A25:01,HLA-A29:02,HLA-B44:03,HLA-B18:01,HLA-C16:01,HLA-C12:03
TCGA.IB.A5ST.01A	HLA-A02:01,HLA-A01:01,HLA-B08:01,HLA-B07:02,HLA-C07:02,HLA-C07:01
TCGA.US.A77E.01A	HLA-A02:01,HLA-A01:01,HLA-B08:01,HLA-B18:01,HLA-C07:01,HLA-C07:01
TCGA.IB.A5SO.01A	HLA-A01:01,HLA-A03:01,HLA-B08:01,HLA-B07:02,HLA-C07:02,HLA-C07:01
TCGA.LB.A8F3.01A	HLA-A36:01,HLA-A26:01,HLA-B08:01,HLA-B07:02,HLA-C07:01,HLA-C03:04
TCGA.HV.A5A5.01A	HLA-A11:01,HLA-A02:01,HLA-B15:01,HLA-B15:01,HLA-C03:03,HLA-C01:02
TCGA.FB.AAQ1.01A	HLA-A02:01,HLA-A03:01,HLA-B15:01,HLA-B07:02,HLA-C03:03,HLA-C07:02
TCGA.IB.A7M4.01A	HLA-A01:01,HLA-A03:01,HLA-B08:01,HLA-B51:01,HLA-C07:01,HLA-C15:02
TCGA.XD.AAUL.01A	HLA-A02:01,HLA-A26:01,HLA-B51:01,HLA-B38:01,HLA-C15:02,HLA-C12:03
TCGA.IB.7649.01A	HLA-A03:01,HLA-A03:01,HLA-B51:01,HLA-B15:01,HLA-C03:04,HLA-C01:02
TCGA.HZ.8317.01A	HLA-A02:01,HLA-A26:01,HLA-B40:01,HLA-B40:01,HLA-C03:04,HLA-C03:04
TCGA.IB.7887.01A	HLA-A30:02,HLA-A25:01,HLA-B15:01,HLA-B18:01,HLA-C05:01,HLA-C03:03
TCGA.2L.AAQE.01A	HLA-A02:01,HLA-A02:01,HLA-B15:01,HLA-B18:01,HLA-C07:01,HLA-C03:03
TCGA.3A.A9IX.01A	HLA-A02:01,HLA-A26:01,HLA-B40:01,HLA-B38:01,HLA-C03:04,HLA-C12:03
TCGA.F2.6879.01A	HLA-A02:01,HLA-A03:01,HLA-B15:01,HLA-B07:02,HLA-C07:02,HLA-C03:03
TCGA.HZ.7920.01A	HLA-A74:01,HLA-A33:03,HLA-B49:01,HLA-B35:01,HLA-C07:01,HLA-C04:01
TCGA.IB.7654.01A	HLA-A02:01,HLA-A03:01,HLA-B57:01,HLA-B35:03,HLA-C06:02,HLA-C04:01

TCGA.IB.AAUV.01A	HLA-A02:05,HLA-A32:01,HLA-B50:01,HLA-B27:05,HLA-C06:02,HLA-C02:02
TCGA.M8.A5N4.01A	HLA-A11:01,HLA-A24:23,HLA-B38:01,HLA-B15:01,HLA-C03:03,HLA-C12:03
TCGA.3A.A9IJ.01A	HLA-A31:01,HLA-A03:01,HLA-B35:01,HLA-B51:01,HLA-C04:01,HLA-C15:02
TCGA.IB.A5SS.01A	HLA-A02:01,HLA-A02:01,HLA-B15:01,HLA-B44:02,HLA-C04:01,HLA-C05:01
TCGA.FB.AAPY.01A	HLA-A24:02,HLA-A11:01,HLA-B35:01,HLA-B07:02,HLA-C07:02,HLA-C04:01
TCGA.S4.A8RP.01A	HLA-A02:01,HLA-A02:01,HLA-B27:05,HLA-B44:03,HLA-C04:01,HLA-C02:02
TCGA.HZ.A9TJ.06A	HLA-A02:01,HLA-A02:01,HLA-B51:01,HLA-B15:01,HLA-C15:02,HLA-C01:02
TCGA.HZ.7919.01A	HLA-A32:01,HLA-A03:01,HLA-B08:01,HLA-B07:02,HLA-C07:02,HLA-C07:01
TCGA.2J.AABP.01A	HLA-A68:02,HLA-A32:01,HLA-B40:02,HLA-B14:02,HLA-C02:02,HLA-C08:02
TCGA.FB.AAQ6.01A	HLA-A03:02,HLA-A32:01,HLA-B49:01,HLA-B40:05,HLA-C07:01,HLA-C03:04
TCGA.2L.AAQI.01A	HLA-A31:01,HLA-A03:01,HLA-B08:01,HLA-B35:01,HLA-C07:01,HLA-C04:01
TCGA.2J.AAB9.01A	HLA-A02:01,HLA-A01:01,HLA-B08:01,HLA-B44:02,HLA-C07:01,HLA-C05:01
TCGA.HV.A5A3.01A	HLA-A24:02,HLA-A33:03,HLA-B58:01,HLA-B55:02,HLA-C03:02,HLA-C03:03
TCGA.IB.AAUR.01A	HLA-A03:01,HLA-A03:01,HLA-B07:02,HLA-B07:02,HLA-C07:02,HLA-C07:02
TCGA.HZ.A77Q.01A	HLA-A68:01,HLA-A03:01,HLA-B08:01,HLA-B15:01,HLA-C07:01,HLA-C03:03
TCGA.2J.AAB8.01A	HLA-A02:01,HLA-A25:01,HLA-B39:01,HLA-B07:02,HLA-C07:02,HLA-C07:02
TCGA.HZ.A8P1.01A	HLA-A02:01,HLA-A03:01,HLA-B40:01,HLA-B35:01,HLA-C03:04,HLA-C04:01
TCGA.2J.AAB4.01A	HLA-A11:01,HLA-A23:01,HLA-B50:01,HLA-B44:02,HLA-C06:02,HLA-C05:01
TCGA.3A.A9IB.01A	HLA-A68:01,HLA-A03:01,HLA-B08:01,HLA-B51:01,HLA-C07:01,HLA-C02:02
TCGA.2J.AABO.01A	HLA-A68:01,HLA-A02:01,HLA-B15:01,HLA-B44:02,HLA-C07:04,HLA-C03:03
TCGA.HZ.8638.01A	HLA-A02:01,HLA-A29:02,HLA-B51:01,HLA-B44:03,HLA-C16:01,HLA-C15:02
TCGA.3E.AAAZ.01A	HLA-A11:01,HLA-A33:01,HLA-B14:02,HLA-B27:05,HLA-C01:02,HLA-C08:02
TCGA.HZ.8003.01A	HLA-A02:01,HLA-A01:01,HLA-B51:01,HLA-B51:01,HLA-C05:01,HLA-C15:02
TCGA.Q3.AA2A.01A	HLA-A02:01,HLA-A01:01,HLA-B08:01,HLA-B13:02,HLA-C07:01,HLA-C06:02
TCGA.2L.AAQA.01A	HLA-A68:01,HLA-A03:01,HLA-B35:01,HLA-B07:02,HLA-C07:04,HLA-C07:02
TCGA.HV.AA8X.01A	HLA-A02:06,HLA-A11:01,HLA-B40:06,HLA-B15:01,HLA-C04:01,HLA-C08:01
TCGA.IB.AAUW.01A	HLA-A30:01,HLA-A01:01,HLA-B13:02,HLA-B57:01,HLA-C06:02,HLA-C06:02
TCGA.F2.A44H.01A	HLA-A31:01,HLA-A33:03,HLA-B50:01,HLA-B44:02,HLA-C06:02,HLA-C05:01
TCGA.3A.A9I5.01A	HLA-A01:01,HLA-A24:02,HLA-B08:01,HLA-B18:01,HLA-C07:01,HLA-C07:01
TCGA.3A.A9I9.01A	HLA-A30:01,HLA-A01:01,HLA-B08:01,HLA-B44:06,HLA-C07:01,HLA-C05:01
TCGA.US.A774.01A	HLA-A24:02,HLA-A11:01,HLA-B18:01,HLA-B18:01,HLA-C07:01,HLA-C07:01
TCGA.IB.A6UF.01A	HLA-A01:01,HLA-A01:01,HLA-B08:01,HLA-B57:01,HLA-C07:01,HLA-C06:02
TCGA.IB.AAUT.01A	HLA-A11:01,HLA-A23:01,HLA-B35:01,HLA-B44:03,HLA-C04:01,HLA-C04:01
TCGA.H6.A45N.01A	HLA-A02:01,HLA-A01:01,HLA-B40:01,HLA-B57:01,HLA-C06:02,HLA-C03:04
TCGA.3A.A9I7.01A	HLA-A74:01,HLA-A02:01,HLA-B15:10,HLA-B44:02,HLA-C04:01,HLA-C05:01
TCGA.HZ.7926.01A	HLA-A68:01,HLA-A02:05,HLA-B49:01,HLA-B53:01,HLA-C07:01,HLA-C04:01
TCGA.RB.A7B8.01A	HLA-A31:01,HLA-A03:01,HLA-B44:02,HLA-B27:05,HLA-C05:01,HLA-C01:02
TCGA.IB.7652.01A	HLA-A02:07,HLA-A11:01,HLA-B40:01,HLA-B46:01,HLA-C07:02,HLA-C01:02
TCGA.2J.AABH.01A	HLA-A01:01,HLA-A25:01,HLA-B08:01,HLA-B40:01,HLA-C07:01,HLA-C03:04
TCGA.3A.A9IL.01A	HLA-A24:02,HLA-A03:01,HLA-B13:02,HLA-B35:01,HLA-C06:02,HLA-C04:01
TCGA.FB.A4P5.01A	HLA-A02:01,HLA-A03:01,HLA-B40:02,HLA-B35:01,HLA-C04:01,HLA-C02:02
TCGA.IB.7890.01A	HLA-A02:01,HLA-A02:01,HLA-B44:02,HLA-B44:02,HLA-C05:01,HLA-C05:01
TCGA.IB.7886.01A	HLA-A02:01,HLA-A01:01,HLA-B08:01,HLA-B07:02,HLA-C07:01,HLA-C07:02
TCGA.HZ.8636.01A	HLA-A31:01,HLA-A01:01,HLA-B08:01,HLA-B40:01,HLA-C07:01,HLA-C03:04
TCGA.HZ.8001.01A	HLA-A02:01,HLA-A03:01,HLA-B15:01,HLA-B44:03,HLA-C16:01,HLA-C03:03
TCGA.IB.7644.01A	HLA-A02:01,HLA-A02:01,HLA-B15:01,HLA-B56:01,HLA-C03:03,HLA-C01:02
TCGA.HV.A5A4.01A	HLA-A24:02,HLA-A02:01,HLA-B40:02,HLA-B40:01,HLA-C03:04,HLA-C04:01
TCGA.2J.AABV.01A	HLA-A03:01,HLA-A03:01,HLA-B49:01,HLA-B15:01,HLA-C01:02,HLA-C07:01
TCGA.IB.AAUN.01A	HLA-A31:01,HLA-A32:01,HLA-B51:01,HLA-B35:01,HLA-C04:01,HLA-C05:01
TCGA.IB.AAUQ.01A	HLA-A02:01,HLA-A01:01,HLA-B40:01,HLA-B44:03,HLA-C03:04,HLA-C04:01
TCGA.2J.AABA.01A	HLA-A23:01,HLA-A03:01,HLA-B40:02,HLA-B44:03,HLA-C16:04,HLA-C04:01

TCGA.XD.AAUH.01A	HLA-A02:01,HLA-A01:01,HLA-B44:02,HLA-B57:01,HLA-C07:02,HLA-C05:01
TCGA.FB.A5VM.01A	HLA-A68:02,HLA-A02:01,HLA-B14:02,HLA-B27:05,HLA-C02:02,HLA-C08:02
TCGA.HZ.A8P0.01A	HLA-A01:01,HLA-A03:01,HLA-B07:02,HLA-B57:01,HLA-C07:02,HLA-C06:02
TCGA.IB.8126.01A	HLA-A68:01,HLA-A03:01,HLA-B40:01,HLA-B38:01,HLA-C03:04,HLA-C12:03
TCGA.PZ.A5RE.01A	HLA-A11:01,HLA-A02:01,HLA-B18:01,HLA-B44:02,HLA-C07:01,HLA-C05:01
TCGA.HZ.7923.01A	HLA-A02:01,HLA-A03:01,HLA-B15:01,HLA-B15:01,HLA-C03:04,HLA-C03:04
TCGA.2J.AABK.01A	HLA-A02:01,HLA-A02:01,HLA-B07:02,HLA-B56:01,HLA-C07:02,HLA-C01:02
TCGA.HZ.A49H.01A	HLA-A02:01,HLA-A02:01,HLA-B15:01,HLA-B07:02,HLA-C07:02,HLA-C03:03
TCGA.2J.AAB6.01A	HLA-A24:02,HLA-A24:02,HLA-B08:01,HLA-B40:01,HLA-C07:01,HLA-C03:04
TCGA.L1.A7W4.01A	HLA-A01:01,HLA-A03:01,HLA-B08:01,HLA-B35:01,HLA-C07:01,HLA-C04:01
TCGA.XN.A8T3.01A	HLA-A01:01,HLA-A26:01,HLA-B08:01,HLA-B39:01,HLA-C07:01,HLA-C12:03
TCGA.HV.A7OP.01A	HLA-A24:02,HLA-A02:01,HLA-B39:05,HLA-B51:01,HLA-C07:02,HLA-C14:02
TCGA.FB.AAPS.01A	HLA-A02:01,HLA-A01:01,HLA-B51:01,HLA-B44:02,HLA-C05:01,HLA-C15:02
TCGA.F2.A7TX.01A	HLA-A11:01,HLA-A03:01,HLA-B35:01,HLA-B35:01,HLA-C04:01,HLA-C04:01
TCGA.2L.AAQJ.01A	HLA-A30:01,HLA-A11:01,HLA-B13:02,HLA-B52:01,HLA-C06:02,HLA-C12:02
TCGA.FB.AAPU.01A	HLA-A30:01,HLA-A02:03,HLA-B13:02,HLA-B38:02,HLA-C07:02,HLA-C06:02
TCGA.XD.AAUG.01A	HLA-A02:01,HLA-A01:01,HLA-B08:01,HLA-B44:02,HLA-C07:01,HLA-C05:01
TCGA.F2.7273.01A	HLA-A02:01,HLA-A01:01,HLA-B44:02,HLA-B15:10,HLA-C05:01,HLA-C02:10
TCGA.FB.AAPZ.01A	HLA-A30:02,HLA-A02:01,HLA-B51:01,HLA-B44:03,HLA-C16:01,HLA-C02:02
TCGA.3A.A9IR.01A	HLA-A31:01,HLA-A02:01,HLA-B27:05,HLA-B52:01,HLA-C12:02,HLA-C02:02
TCGA.LB.A9Q5.01A	HLA-A02:01,HLA-A02:01,HLA-B40:01,HLA-B07:02,HLA-C07:02,HLA-C03:04
TCGA.Q3.A5QY.01A	HLA-A02:01,HLA-A32:01,HLA-B52:01,HLA-B15:01,HLA-C12:02,HLA-C03:04
TCGA.IB.A5SP.01A	HLA-A24:02,HLA-A26:01,HLA-B51:01,HLA-B18:01,HLA-C07:01,HLA-C01:02
TCGA.IB.7897.01A	HLA-A24:02,HLA-A01:01,HLA-B51:01,HLA-B57:01,HLA-C06:02,HLA-C03:03
TCGA.3A.A9IU.01A	HLA-A02:01,HLA-A01:01,HLA-B08:01,HLA-B44:02,HLA-C07:01,HLA-C05:01
TCGA.F2.A8YN.01A	HLA-A02:01,HLA-A03:01,HLA-B08:01,HLA-B08:01,HLA-C07:01,HLA-C07:01
TCGA.FB.AAPQ.01A	HLA-A29:02,HLA-A03:01,HLA-B07:02,HLA-B44:03,HLA-C16:01,HLA-C12:02
TCGA.YB.A89D.01A	HLA-A02:01,HLA-A03:01,HLA-B14:01,HLA-B15:01,HLA-C03:04,HLA-C08:02
TCGA.FB.A7DR.01A	HLA-A02:01,HLA-A33:03,HLA-B40:02,HLA-B18:01,HLA-C07:01,HLA-C15:02
TCGA.2J.AABT.01A	HLA-A11:01,HLA-A11:01,HLA-B35:01,HLA-B51:01,HLA-C04:01,HLA-C14:02
TCGA.HZ.7924.01A	HLA-A02:01,HLA-A03:01,HLA-B51:01,HLA-B52:01,HLA-C15:02,HLA-C12:02
TCGA.2J.AAB1.01A	HLA-A02:01,HLA-A26:01,HLA-B08:01,HLA-B14:01,HLA-C07:01,HLA-C08:02
TCGA.IB.7888.01A	HLA-A24:02,HLA-A33:03,HLA-B39:06,HLA-B52:01,HLA-C07:02,HLA-C16:01
TCGA.IB.AAUM.01A	HLA-A02:01,HLA-A26:01,HLA-B08:01,HLA-B40:01,HLA-C07:01,HLA-C03:04
TCGA.HV.A7OL.01A	HLA-A31:01,HLA-A02:01,HLA-B46:01,HLA-B15:01,HLA-C03:03,HLA-C01:02
TCGA.IB.7646.01A	HLA-A02:01,HLA-A32:01,HLA-B27:02,HLA-B44:02,HLA-C05:01,HLA-C02:02
TCGA.HV.A5A6.01A	HLA-A02:06,HLA-A02:07,HLA-B46:01,HLA-B51:01,HLA-C14:02,HLA-C01:02
TCGA.3A.A9J0.01A	HLA-A01:01,HLA-A32:01,HLA-B44:02,HLA-B57:01,HLA-C06:02,HLA-C05:01
TCGA.2L.AAQM.01A	HLA-A02:01,HLA-A02:01,HLA-B35:01,HLA-B51:01,HLA-C04:01,HLA-C14:02
TCGA.HZ.8519.01A	HLA-A02:01,HLA-A01:01,HLA-B08:01,HLA-B08:01,HLA-C07:01,HLA-C07:01
TCGA.3A.A9IN.01A	HLA-A24:02,HLA-A03:01,HLA-B07:02,HLA-B18:01,HLA-C07:02,HLA-C12:03
TCGA.US.A77J.01A	HLA-A24:02,HLA-A26:01,HLA-B14:02,HLA-B38:01,HLA-C12:03,HLA-C02:02
TCGA.IB.AAUS.01A	HLA-A23:01,HLA-A01:01,HLA-B08:01,HLA-B44:03,HLA-C07:01,HLA-C04:01
TCGA.US.A779.01A	HLA-A24:02,HLA-A11:01,HLA-B51:08,HLA-B55:01,HLA-C16:02,HLA-C03:03
TCGA.HZ.7925.01A	HLA-A29:02,HLA-A03:01,HLA-B56:01,HLA-B58:01,HLA-C07:01,HLA-C01:02
TCGA.FB.A4P6.01A	HLA-A02:01,HLA-A01:01,HLA-B08:01,HLA-B40:01,HLA-C07:01,HLA-C03:04
TCGA.IB.7893.01A	HLA-A02:01,HLA-A01:01,HLA-B08:01,HLA-B44:02,HLA-C07:01,HLA-C05:01
TCGA.FB.AAQ0.01A	HLA-A01:01,HLA-A32:01,HLA-B08:01,HLA-B44:02,HLA-C07:01,HLA-C05:01
TCGA.FB.AAQ3.01A	HLA-A68:01,HLA-A29:02,HLA-B44:04,HLA-B07:02,HLA-C07:02,HLA-C16:01
TCGA.2J.AABU.01A	HLA-A24:02,HLA-A33:01,HLA-B07:02,HLA-B15:07,HLA-C07:02,HLA-C01:02
TCGA.2L.AAQL.01A	HLA-A02:01,HLA-A01:01,HLA-B51:01,HLA-B58:01,HLA-C07:01,HLA-C15:02

TCGA.IB.7889.01A	HLA-A24:02,HLA-A68:01,HLA-B14:01,HLA-B07:02,HLA-C07:02,HLA-C08:02
TCGA.FB.A545.01A	HLA-A02:01,HLA-A02:01,HLA-B27:05,HLA-B57:01,HLA-C06:02,HLA-C02:02
TCGA.HZ.A77O.01A	HLA-A01:01,HLA-A26:01,HLA-B52:01,HLA-B35:03,HLA-C04:01,HLA-C12:02
TCGA.RB.AA9M.01A	HLA-A30:01,HLA-A11:01,HLA-B13:02,HLA-B35:01,HLA-C06:02,HLA-C04:01
TCGA.HZ.8315.01A	HLA-A29:02,HLA-A33:03,HLA-B15:16,HLA-B44:03,HLA-C16:01,HLA-C14:02
TCGA.IB.7885.01A	HLA-A02:01,HLA-A29:02,HLA-B41:01,HLA-B44:03,HLA-C17:01,HLA-C16:01
TCGA.HZ.8002.01A	HLA-A69:01,HLA-A11:01,HLA-B15:17,HLA-B52:01,HLA-C07:01,HLA-C12:02
TCGA.S4.A8RM.01A	HLA-A02:01,HLA-A03:01,HLA-B40:01,HLA-B44:02,HLA-C03:04,HLA-C05:01
TCGA.Z5.AAPL.01A	HLA-A25:01,HLA-A24:02,HLA-B07:02,HLA-B18:01,HLA-C07:02,HLA-C12:03
TCGA.US.A77G.01A	HLA-A01:01,HLA-A03:01,HLA-B35:01,HLA-B57:01,HLA-C06:02,HLA-C04:01
TCGA.3A.A9IC.01A	HLA-A24:02,HLA-A02:01,HLA-B50:01,HLA-B27:05,HLA-C06:02,HLA-C02:02
TCGA.3A.A9IO.01A	HLA-A02:01,HLA-A01:01,HLA-B51:01,HLA-B44:02,HLA-C06:02,HLA-C05:01
TCGA.FB.AAPP.01A	HLA-A02:01,HLA-A03:01,HLA-B15:01,HLA-B07:02,HLA-C03:03,HLA-C07:02
TCGA.IB.7651.01A	HLA-A03:01,HLA-A01:01,HLA-B08:01,HLA-B07:02,HLA-C07:02,HLA-C07:01
TCGA.XD.AAUI.01A	HLA-A29:02,HLA-A02:01,HLA-B07:02,HLA-B44:03,HLA-C07:02,HLA-C16:01
TCGA.3E.AAAY.01A	HLA-A68:01,HLA-A03:01,HLA-B07:05,HLA-B35:02,HLA-C15:05,HLA-C04:01
TCGA.IB.AAUO.01A	HLA-A02:01,HLA-A01:01,HLA-B08:01,HLA-B15:01,HLA-C07:01,HLA-C04:01
TCGA.H6.8124.01A	HLA-A31:01,HLA-A01:01,HLA-B08:01,HLA-B39:01,HLA-C07:01,HLA-C12:03
TCGA.F2.6880.01A	HLA-A02:01,HLA-A01:01,HLA-B08:01,HLA-B27:05,HLA-C07:01,HLA-C02:02
TCGA.HZ.8637.01A	HLA-A02:01,HLA-A01:01,HLA-B08:01,HLA-B15:01,HLA-C07:01,HLA-C03:04
TCGA.YH.A8SY.01A	HLA-A23:01,HLA-A03:01,HLA-B44:03,HLA-B18:01,HLA-C04:01,HLA-C12:03
TCGA.IB.8127.01A	HLA-A29:02,HLA-A32:01,HLA-B45:01,HLA-B44:03,HLA-C06:02,HLA-C16:01
TCGA.2J.AABF.01A	HLA-A24:02,HLA-A32:01,HLA-B40:01,HLA-B44:02,HLA-C07:04,HLA-C03:04
TCGA.HZ.8005.01A	HLA-A26:01,HLA-A32:01,HLA-B15:01,HLA-B38:01,HLA-C12:03,HLA-C03:03
TCGA.3A.A9IV.01A	HLA-A30:02,HLA-A03:01,HLA-B07:02,HLA-B18:01,HLA-C07:02,HLA-C05:01
TCGA.IB.7645.01A	HLA-A24:02,HLA-A24:02,HLA-B40:02,HLA-B38:01,HLA-C03:04,HLA-C12:03
TCGA.IB.AAUP.01A	HLA-A24:02,HLA-A02:01,HLA-B13:02,HLA-B15:01,HLA-C06:02,HLA-C01:02
TCGA.2J.AABR.01A	HLA-A02:01,HLA-A02:01,HLA-B40:01,HLA-B51:01,HLA-C03:04,HLA-C02:02
TCGA.3A.A9IZ.01A	HLA-A29:02,HLA-A01:01,HLA-B44:02,HLA-B44:03,HLA-C05:01,HLA-C16:01
TCGA.HZ.7922.01A	HLA-A31:01,HLA-A01:01,HLA-B07:02,HLA-B51:07,HLA-C07:02,HLA-C14:02
TCGA.2J.AABI.01A	HLA-A02:01,HLA-A03:01,HLA-B44:02,HLA-B44:02,HLA-C05:01,HLA-C05:01
TCGA.2J.AABE.01A	HLA-A01:01,HLA-A01:01,HLA-B08:01,HLA-B44:03,HLA-C07:01,HLA-C16:01
TCGA.HZ.A49I.01A	HLA-A68:01,HLA-A02:01,HLA-B44:02,HLA-B44:02,HLA-C07:04,HLA-C05:01
TCGA.HZ.A4BK.01A	HLA-A01:01,HLA-A29:02,HLA-B44:03,HLA-B18:01,HLA-C16:01,HLA-C05:01
TCGA.HZ.A4BH.01A	HLA-A01:01,HLA-A25:01,HLA-B08:01,HLA-B44:03,HLA-C07:01,HLA-C16:01
TCGA.HZ.A77P.01A	HLA-A02:01,HLA-A32:01,HLA-B40:01,HLA-B51:01,HLA-C03:04,HLA-C01:02
TCGA.HZ.7918.01A	HLA-A23:01,HLA-A02:01,HLA-B39:01,HLA-B13:01,HLA-C12:03,HLA-C04:03
TCGA.3A.A9IH.01A	HLA-A29:02,HLA-A02:01,HLA-B08:01,HLA-B45:01,HLA-C07:01,HLA-C06:02
TCGA.US.A776.01A	HLA-A68:01,HLA-A01:01,HLA-B08:01,HLA-B40:01,HLA-C07:01,HLA-C03:04
TCGA.IB.A5SQ.01A	HLA-A68:01,HLA-A30:01,HLA-B13:02,HLA-B44:02,HLA-C07:04,HLA-C06:02
TCGA.F2.A44G.01A	HLA-A01:01,HLA-A03:01,HLA-B08:01,HLA-B35:01,HLA-C07:01,HLA-C04:01
TCGA.LB.A7SX.01A	HLA-A33:03,HLA-A03:01,HLA-B15:16,HLA-B58:02,HLA-C06:02,HLA-C14:02
TCGA.RL.AAAS.01A	HLA-A11:01,HLA-A03:01,HLA-B35:01,HLA-B07:22,HLA-C07:02,HLA-C04:01
TCGA.IB.A6UG.01A	HLA-A02:01,HLA-A03:01,HLA-B08:01,HLA-B45:01,HLA-C07:01,HLA-C06:02
TCGA.HV.AA8V.01A	HLA-A02:06,HLA-A33:03,HLA-B46:01,HLA-B44:03,HLA-C07:01,HLA-C01:02
TCGA.H8.A6C1.01A	HLA-A01:01,HLA-A32:01,HLA-B15:17,HLA-B35:02,HLA-C07:01,HLA-C04:01

Table S2. neoTST that detected in PDAC plasma EVs

ID	type	sample counts in PDAC tissue	sample counts in PDAC plasma Evs
chr17:16382339|16382568:-	E-E-AS	69	184
chr12:124912883|124913112:+	intergenic	47	171
chr11:308344|314263:-	intergenic	3	170
chr1:55178129|55214790:-	E-E-AS	3	116
chr6:29934253|29943510:-	intergenic	13	111

Table S3. PDAC neoTSTs and their derived neoantigen for figure 6

MHC	Peptide	Identity	%Rank_EL	%Rank_BA	Aff(nM)	Bind Level	lable	Num
HLA-A*11:01	STHGAVNTSK	2_PARN	0.06	0.21	33.99	<=	SB	hum1
HLA-A*11:01	TSQVYKNWSVK	3_chr2	1.78	1.2	295.73	<=	WB	hum2
HLA-A*11:01	ATSTSQVYKN	3_chr2	0.66	0.38	64.1	<=	WB	hum2
HLA-A*11:01	SQVYKNWSVK	3_chr2	0.73	0.34	56.49	<=	WB	hum2
HLA-A*11:01	QQNRFTMMY	3_chr2	1.14	1.57	462.15	<=	WB	hum2
HLA-A*11:01	FTMMYACCK	3_chr2	1.6	0.15	24.51	<=	WB	hum2
HLA-A*11:01	SASASWLTR	3_chr2	0.56	0.75	142.21	<=	WB	hum2
HLA-A*11:01	LTATSTSQVYK	3_chr2	0.35	0.18	29.56	<=	SB	hum2
HLA-A*11:01	TATSTSQVYK	3_chr2	0.13	0.06	13.38	<=	SB	hum2
HLA-A*11:01	RTIDGVSLY	3_chr2	0.04	0.22	35.66	<=	SB	hum2
HLA-A*11:01	ATSTSQVYK	3_chr2	0	0.01	4.82	<=	SB	hum2
HLA-A*11:01	QVYKNWSVK	3_chr2	0.19	0.3	48.63	<=	SB	hum2
HLA-A*02:01	FPGMAGQTPGI	3_chr2	1.73	1.4	148.65	<=	WB	hum2
HLA-A*02:01	RILLYHPAGV	3_chr2	1.82	0.89	76.69	<=	WB	hum2
HLA-A*02:01	SLYCPGWRAV	3_chr2	0.88	0.41	27.95	<=	WB	hum2
HLA-A*02:01	VQWHHCSLL	3_chr2	1.98	2.48	402.03	<=	WB	hum2
HLA-A*02:01	WLTRTIDGV	3_chr2	1.83	0.55	40.04	<=	WB	hum2
HLA-A*02:01	SLYCPGWRA	3_chr2	0.69	0.66	50.64	<=	WB	hum2
HLA-A*02:01	GMAGQTPGI	3_chr2	0.28	0.66	50.5	<=	SB	hum2
HLA-A*02:01	ILLYHPAGV	3_chr2	0.13	0.12	9.25	<=	SB	hum2
HLA-A*02:01	SQVYKNWSV	3_chr2	0.4	0.58	43.32	<=	SB	hum2
HLA-A*11:01	ASFPCCSCR	4_STRG	1.55	0.95	204.58	<=	WB	hum3
HLA-A*11:01	ASGHLDLSK	4_STRG	0.3	0.5	85.65	<=	SB	hum3
HLA-A*02:01	RPVIGVISAV	4_STRG	1.97	1.51	168.6	<=	WB	hum3
HLA-A*02:01	SLSGVIWYP	4_STRG	1.21	1.74	214.38	<=	WB	hum3
HLA-A*02:01	LLANANDGL	4_STRG	1.16	1.01	89.51	<=	WB	hum3
HLA-A*02:01	VIWYPTPSL	4_STRG	0.35	1.65	195.86	<=	SB	hum3
HLA-A*11:01	ASLTPPRARR	5_STRG	0.63	1.59	472.94	<=	WB	hum4
HLA-A*11:01	ASAQPGPAQR	5_STRG	0.24	1.19	289.2	<=	SB	hum4
HLA-A*11:01	RLASLTPPR	5_STRG	0.43	0.5	85.63	<=	SB	hum4
HLA-A*11:01	ASLTPPRAR	5_STRG	0.26	1.36	360.77	<=	SB	hum4
HLA-A*02:01	RLASLTPPRA	5_STRG	1.6	1.5	167.78	<=	WB	hum4
HLA-A*02:01	SLPGPAHRL	5_STRG	0.21	2.74	479.93	<=	SB	hum4
HLA-A*11:01	ISVDKVKAFYK	6_chr2	0.64	0.35	58.58	<=	WB	hum5
HLA-A*11:01	HTIISVDKVK	6_chr2	1	0.97	210.16	<=	WB	hum5
HLA-A*11:01	YKIQHPFMIK	6_chr2	1.05	0.43	74.16	<=	WB	hum5
HLA-A*11:01	KQGTEGIYLK	6_chr2	0.59	0.97	211.36	<=	WB	hum5
HLA-A*11:01	AIYDIPTAKV	6_chr2	0.81	1.56	459.34	<=	WB	hum5
HLA-A*11:01	TIISVDKVK	6_chr2	0.84	1.42	386.28	<=	WB	hum5
HLA-A*11:01	IRAIYDIPTAK	6_chr2	0.3	0.86	174.39	<=	SB	hum5
HLA-A*11:01	SVDKVKAFYK	6_chr2	0.14	0.04	10.6	<=	SB	hum5
HLA-A*11:01	RAIYDIPTAK	6_chr2	0.02	0.14	23.19	<=	SB	hum5
HLA-A*11:01	GTAPVYSSQR	6_chr2	0.22	0.65	116.53	<=	SB	hum5
HLA-A*11:01	MIYHISRIK	6_chr2	0.41	0.28	44.98	<=	SB	hum5
HLA-A*11:01	KIQHPFMIK	6_chr2	0.03	0.05	12.3	<=	SB	hum5
HLA-A*11:01	AIYDIPTAK	6_chr2	0	0.02	8.7	<=	SB	hum5
HLA-A*02:01	LVSPLLFNI	6_chr2	0.67	0.54	38.38	<=	WB	hum5

HLA-A*02:01	AIYDIPTAKV	6_chr2	0.15	1.13	106.91	<=	SB	hum5
HLA-A*11:01	ASQIGVLGIR	7_chr8	1.44	1.61	483.29	<=	WB	hum6
HLA-A*11:01	MQVSFWFMIK	10_RAS/	1.71	0.11	19.12	<=	WB	hum7
HLA-A*11:01	QVSFWFMIKR	10_RAS/	1.94	0.67	122.63	<=	WB	hum7
HLA-A*11:01	VSFWFMIKR	10_RAS/	0.56	0.28	44.33	<=	WB	hum7
HLA-A*11:01	QVSFWFMIK	10_RAS/	0.29	0.05	12.18	<=	SB	hum7
HLA-A*02:01	GIQNFPEGL	11_SLC 39A11	1.39	5.92	2078.57	<=	WB	hum8
HLA-A*02:01	TCMGPVVPLV	te01	1.4	1.12	105.8	<=	WB	hum9
HLA-A*02:01	QLPRGVEPV	te01	0.84	1.57	180.1	<=	WB	hum9
HLA-A*02:01	WMSKWKFTV	te01	0.58	0.05	5.82	<=	WB	hum9
HLA-A*02:01	ALPCKATGV	te01	1.3	1.84	235.67	<=	WB	hum9
HLA-A*02:01	LLHQHDLDV	te01	1.13	2.58	428.82	<=	WB	hum9
HLA-A*02:01	SLHHGLEKA	te01	0.39	2.04	286.24	<=	SB	hum9
HLA-A*02:01	CMGPVVPLV	te01	0.41	0.21	15.36	<=	SB	hum9
HLA-A*11:01	STIEVWEPPPR	te01	0.63	1.1	252.03	<=	WB	hum9
HLA-A*11:01	CIPATPAVTK	te01	0.86	0.49	83.82	<=	WB	hum9
HLA-A*11:01	AMASEGASPK	te01	0.75	0.53	92.28	<=	WB	hum9
HLA-A*11:01	ASPKPWQLPR	te01	0.89	1.13	263.7	<=	WB	hum9
HLA-A*11:01	RMYRNAWMSK	te01	0.57	0.05	12.84	<=	WB	hum9
HLA-A*11:01	WRTSARSIQK	te01	1.37	0.87	179.07	<=	WB	hum9
HLA-A*11:01	SQAWRLRKK	te01	0.62	0.83	166.03	<=	WB	hum9
HLA-A*11:01	MASEGASPK	te01	0.67	0.48	82.49	<=	WB	hum9
HLA-A*11:01	SWRTSARSIQK	te01	0.32	0.49	83.43	<=	SB	hum9
HLA-A*11:01	STDSLHHGLEK	te01	0.11	0.21	33.74	<=	SB	hum9
HLA-A*11:01	RGVEPVGAQK	te01	0.21	1.08	247.35	<=	SB	hum9
HLA-A*11:01	AVIRGPPSSR	te01	0.18	1.12	263.09	<=	SB	hum9
HLA-A*11:01	TDSLHHGLEK	te01	0.49	0.39	67.44	<=	SB	hum9
HLA-A*11:01	ATDTQCQPVK	te01	0.34	0.45	76.51	<=	SB	hum9
HLA-A*11:01	GVEPVGAQK	te01	0.14	0.93	199.33	<=	SB	hum9
HLA-A*11:01	RTSARSIQK	te01	0.02	0.06	13.28	<=	SB	hum9
HLA-A*11:01	KATGVELPK	te01	0.31	0.43	73.37	<=	SB	hum9
HLA-A*11:01	KTIGTHLLH	te01	0.26	0.73	136.66	<=	SB	hum9

Table S4. neoTST and mutation in KPC for validation

Num	NeoTST	Type
neoTST1	1:54651760|54653061:-	Intron_AS
neoTST2	7:113706129|1137066790:-	E-E
neoTST3	2:78347649|783544435:-	intergenic-intergenic
neoTST4	2:14279748|14279836:+	Intron_AS
**Num**	**Mutation**	**Gene/Protein Anno**
mut1	11:60779106	Smcr8/p.V360A
mut2	11:67856506	Usp43/p.G787S
mut3	11:69828394	Nlgn2/p.V210A
mut4	8:91090175	Rbl2/p.S431T
mut5	4:16132819	Ripk2/p.D299E

Table S5. Panc02 mutation neoantigen sequence

Num	Name	Sequence
1	Arfgef1	WLCAVSMDELLSATHPRMFSLQKIV
2	Cant1	LDTGSRAQEENTLFSYLKKGYLTLS
3	Clasp1	QKALQSHLKNSDNIVSLPQSDRSSS
4	Dpy19l1	LIALCISNVLFMVPWQFAQFVLLTQ
5	Dtx3l	LSKEQRASITTVYPHIIKSMEGSDG
6	Fbf1	EKFSSSLNTLSSLVEASHLTTSQQR
7	Klhl7	PLISKNFLSKTVLAEPLIQDNPECL
8	Nfat5	NTVQQHPSTPKRYTVLYISPPPEDL
9	Pcnx	YSDSHDQLKEILEGPISLGNIRNFI
10	Pdpr	SAASQNGVQIYDWTSVLHVLIKKGQ
11	Prrc2b	WMMMPSYMDPRISPTRTPVDFYPSA
13	Rab11fip5	EGPVPRPHNSISYTLLSQKVLGTSE
14	Slc16a13	TGFFIPYVHLVANLQDLGWDPLPAA
15	Snapc4	APDPVQRAPSPDEVSAPSPLDASDG
16	Tep1	EAGQQLGQFSRHQSAVSAVVAVEEH
17	Vps13b	SWAWSFVPAIVSYDDVEEDDLGTDP
18	Zfp938	FQCKHCSKTFSYPSSLHMHEKNHTG

Table S6. Panc02 neoTST sequence

Num	Name	Sequence
mus1	2:78355433|78412822:-	MPSDSKFVICSSLPLRTLLFKSCRSRQLAPSLGLEY EGTSEDNHSVLCGRHLAQEPAASTRDPSLPFSKYS QEEIWKQSTEHIVKERLSRDCSTWGSIPYTATKPR CYCGCWEGFPDKSLIWLSPERLCQRRSSQSTIGLS SGVPDRGVGEGTERHYVLRENHAM
mus3	16:24733772|24761593:+	MSQRALKSFTLFGSASSIASPPVST
mus5	X:18277550|18278626:+	LMQVYDQFTLVSSSITIRLILILFH
mus2	11:95068839|95080791:-	MLMENTKKALLLTGWDSKAWGRKVALLSLLAG

mus4	17:32373213:32374635:+	MWIHSRISPKRNILPYHEAVAQMIITGKRQALTYFGKEPDIIVQPYSMSQDTWLKQHSTNWLLAQLGFEGTLDSHYPQDRLIKFLNVHDMIFPKMTSLQPLNNALLIFTDGSSKGRAGYLISNQQVIVETPGLSAQLAELTAILKVFQSVHEAFNIFTDSLYVAQSVPLLETCGTFNFNTPSGSLFSELQNIILARKNPFYIGHIRSHSGLPGPLAEGNDRIDRALIGEALVSDRVALAQRDHERFHLSSHTLRLRHKITKEQARMIVKQCPKCITLSPVPHLGVNPRGLMPNHIWQMDITHYTEFGKLKYIHVCIDTCSGFLFASLHTGEASKNVIDHCLQAFNAMGLPKLIKTDNGPSYSSKNFISFCKEFGIKHKTGIPYNPMGQGIVERAHRTLKNWLFKTKEGQLYPPRSPKAHLAFTLFVLNFLHTDIKGQSAADRHWHPVTSNSYALVKWKDPLTNKWKGPDPVLL

Table S7. PDAC neoTST sequence

Num	Name	Sequence
hum1	chr16:14482827|14511216:-;PARN	MRPWTHHPVSTHGAVNTSKYAESYRIQTYA
hum2	chr2:223720994|223723858:-;NA	MCVCVLNFPGMAGQTPGIYHRQQNRFTMMYACCKGRDRILLYHPAGVQWHHCSLLQPQTPGSRDPSASASWLTRTIDGVSLYCPGWRAVAGSWLTATSTSQVYKNWSVKLVFQLVLDANHDLPWPVACSAPQSI
hum3	chr9:133861432|133888891:-;VAV2	MTLVSWTRNPGQDLGVSLSGVIWYPTPSLWFAGPRFPTPRAEEQAALRPLSAQLQIPAGPASGHLDLSKELLRSEASPGTGTRCPCPPGRGASFPCCSCRLLANANDGLSARAQRPVIGVISAVSRLSLHSIAQNK
hum4	chr19:50723628|50724010:+;CLEC11A	MGLGEQRGSGREAGEEEEEEATPTPSSGPSPSPTPEDIVTYILGRLAGLDAGLHQLHVRLHALDTRVVELTQGLRQLRNAAGDTRDAVQALQEAQGRAEREHGRLEDSECMTPPPSLPGPAHRLPEGAAPGPQVLPALARLRSSGGGAGAVHGAGREPGAAGRPPADGGAHSVPARGARSLQLARVAGRARSARRGPLPLRKRPARVLLRLASLTPPRARRPAQRLAASAQPGPAQRWHARELRGAGL
hum5	chr2:134550661|134656477:-;TMEM163	MIYHISRIKNKNHTIISVDKVKAFYKIQHPFMIKTLNKQGTEGIYLKIIRAIYDIPTAKVILSGQRLQPFPLRTGTRQGCLVSPLLFNIAFDDLARAVSQEKEIEGGGRSQDGRIGTAPVYSSQRERRRRRVISAFPSEFDAILDVLSSAIVLWRY
hum6	chr8:30501578|30504286:+;RBPMS	MEPAFVETVGCPVASQIGVLGIRFDPEIPQTLRLEFA
hum7	chr13:114000932|114004804:-;RASA3	MPRFLYPVVRWWTRGFRVFAVVSGSAVNVRMQVSFWFMIKRAQGRKRFGMKNF
hum8	chr17:72649268|72717746:-;SLC39A11	MLKSCTPERNLAIGIGIQNFPEGLA

hum9	chr15:70592041:70592905:+;TE	MSNEEPNVNPQNNGENVSRACQRSSQQPLPSQAWRLRKKTWFHEPGPGSWCCVQPRDLVPCIPATPAVTKRIQGTAWAMASEGASPKPWQLPRGVEPVGAQKSTIEVWEPPPRFQRMYRNAWMSKWKFTVGAGPSWRTSARSIQKGNVGLEPPHTVPTGAPPSGAVIRGPPSSRPQNGRSTDSLHHGLEKATDTQCQPVKAARREALPCKATGVELPKTIGTHLLHQHDLDVRHGVKGDHSGALRFDCPAGFWTCMGPVVPLVWPISPIWNGCIYPMPVLPLYLGSN
